# Quality Control of Shenqi Tongmai Oral Liquid Based on Quantitative Analysis of Multicomponents by Single Marker, Molecular Docking, and Multivariate Statistics

**DOI:** 10.1002/pca.3520

**Published:** 2025-02-19

**Authors:** Qian Li, Li Wang, Liuping Tan, Xiaojing Tao, Bei Zhang, Jingnan Pei, Qiuping Li

**Affiliations:** ^1^ Department of Pharmacy Liuzhou Hospital of Traditional Chinese Medicine Liuzhou China; ^2^ Graduate School of Guangxi University of Traditional Chinese Medicine Nanning China

**Keywords:** antioxidant, comprehensive quality control, molecular docking, Shenqi Tongmai oral liquid (SQTM), spectrum‐effect relationship

## Abstract

**Introduction:**

Shenqi Tongmai oral liquid (SQTM) is famous for its remarkable effect in the treatment of cardiovascular diseases. However, the SQTM quality evaluation system has not been established.

**Objective:**

The objective of this study is to establish a method for the determination of nine components of SQTM and to screen quality control indicators to comprehensively evaluate the quality of SQTM.

**Methods:**

The fingerprints of SQTM were established, and the contents of nine components in 17 batches of SQTM were determined based on quantitative analysis of multicomponents by single marker (QAMS). The antioxidant activity of samples was determined by the DPPH method and hydroxyl method, and the correlation between the content of nine components and antioxidant activity was analyzed by gray relational analysis (GRA), bivariate correlation analysis (BCA), and partial least squares regression (PLSR). The antioxidant activity of the monomers was confirmed through molecular docking techniques and in vitro experiments.

**Results:**

There was no significant difference in the content between the QAMS method and the external standard method (*p* > 0.05). The findings from multivariate statistics, molecular docking, and in vitro validation indicated that rosmarinic acid, luteolin, and protocatechualdehyde exhibited significant antioxidant activities, which were important pharmacodynamic components that exerted antioxidant effects and could serve as quality markers (Q‐Markers).

**Conclusion:**

The study elucidated the Q‐Markers of SQTM and provided a relatively comprehensive approach for the assay of SQTM, which is a promising advance in the quality control of SQTM.

## 1 Introduction

Cardiovascular diseases (CVDs) exhibit high morbidity and mortality rates worldwide [1]. CVD deaths account for 33% of the total global deaths, and from 1990 to 2019, the incidence of CVD ranged from 271 million to 523 million [2]. The incidence of cardiovascular diseases with atherosclerosis as the pathological basis has been increasing year by year, and between 2010 and 2030, the incidence of cardiovascular events will increase by more than 50% per year, demonstrating a gradually younger demographic trend and posing a severe threat to human health [3].

Shenqi Tongmai oral liquid (SQTM) is composed of *Ginseng radix et rhizoma rubra*, *Codonopsis radix*, *Astragali radix*, *Salviae miltiorrhizae radix et rhizoma*, *Ophiopogonis radix*, and *Schisandrae chinensis fructus*. It has the effects of tonifying blood and qi, nourishing yin, and promoting fluid production, and the clinical application in the treatment of cardiovascular diseases has a significant effect.

Clarifying the quality standard of SQTM is conductive to controlling the overall internal quality of traditional Chinese medicine (TCM) preparations throughout the entire process, thereby ensuring the stability of clinical efficacy. At present, the relevant quality standards and evaluation studies of SQTM have not been established, and the correlation evaluation of the active components and antioxidant activity in SQTM has not been carried out. The research on a single chemical quality is difficult to comprehensively reflect the multicomponent characteristics of SQTM and cannot fully demonstrate the overall evaluation of TCM preparations. Moreover, due to the synergistic effects of components, targets and pathways, the determination of Q‐Markers may have no relation with clinical efficacy, making it hard to guarantee the stability and reliability of clinical efficacy. Nevertheless, according to the research of Wang et al. [4], who successfully applied fingerprint chromatogram, QAMS, and spectrum‐effect relationship to the comprehensive quality research and active ingredient exploration of Zuojin Pill, it is learned that multilevel quality control can ensure the stability and consistency of clinical efficacy. This comprehensive quality control strategy is conducive to improving the quality standards of TCM preparations, ensuring their safety, efficacy and stability, and promoting the modernization and globalization of TCM. Therefore, multilevel quality control of TCM preparations is particularly important [5].

The spectrum‐effect relationship integrates the fingerprint with the efficacy. Through certain chemometric methods, a mathematical model of the spectrum‐effect relationship is established. Thus, the group of compounds related to efficacy is identified, establishing a quality evaluation method that reflect the quality of TCM preparations. It can comprehensively reflect the pharmacodynamic substance foundation of TCM preparations as a whole, providing a basis for the establishment of quality markers. Consequently, the quality control indicators become more targeted, reflecting the internal quality of the consistency between TCM preparations and their efficacy [6]. The method of quantitative analysis of multicomponents by single marker (QAMS) involves utilizing a specific component as an internal reference, and through the calculation of relative correction factors (RCFs), it enables simultaneous quantitative analysis of multiple components. Among them, the RCF is a scale factor used to measure the difference in the degree of response of different components to be tested under a particular analytical method. The QAMS method offers advantages such as cost savings and simplicity in operation, making it suitable for the multi‐indicator quality control model of TCM preparations [7]. Molecular docking has emerged as a crucial means for screening potential Q‐Markers with specific activity [8]. Gray relational analysis (GRA), based on the gray process within gray systems, provides an intuitionistic representation of the comprehensive evaluation values for various indicators. It measures the degree of correlation between factors according to the similarity or dissimilarity in their developmental trends and ultimately evaluates the indicators to be tested using the relative degree of correlation [9]. Bivariate correlation analysis (BCA) can establish a correlation between pharmacological effects and the concentration of each chemical component, identify key active components responsible for these effects, and provide guidance for further screening and in‐depth research on active constituents [10]. Partial least squares regression (PLSR) analysis can integrate multiple regression analysis, canonical correlation analysis, and principal component analysis to simplify data; analyze the correlation between variables; and establish regression models. It offers advantages such as reduced computational complexity and high prediction accuracy [11]. Oxidative stress can lead to overexpression of reactive oxygen species (ROS), which promotes abnormal proliferation and migration of vascular smooth muscle cells. It can also initiate lipid peroxidation and endothelial cell membrane damage and thereby participate in the occurrence and development of cardiovascular diseases. Evaluating the antioxidant activity of potential pharmacodynamic components based on their ability to scavenge free radicals in vitro can further identify quality markers for SQTM [12].

In this paper, SQTM was taken as the research subject, and the contents of eight components were determined simultaneously by the QAMS method. Based on the correlation evaluation of antioxidant activity and component indicators of multicomponent by PLSR, GRA, and BCA, the quality markers with antioxidant activity were preliminarily screened. Further molecular docking and monomer verification of antioxidant activity were performed on candidate ingredients to determine reasonable quality markers, thereby comprehensively evaluating their quality. The purpose of this paper is to provide a reference for the overall quality improvement and consistent evaluation of the curative effect of SQTM and to furnish an effective, objective, and feasible method for the comprehensive quality evaluation of SQTM.

## 2 Materials and Methods

### 2.1 Reagents and Materials

Shenqi Tongmai oral liquid (Batch Numbers 20210303, 20220101, 20220302, 20230201, 20230202, 20230301, 20230302, 20230303, 20230304, 20230401, 20230402, 20230501, 20230601, 20230602, 20230701, 20230801, and 20231001) is provided by the Preparation Room of Liuzhou Traditional Chinese Medical Hospital; *Ginseng radix et rhizoma rubra* (Batch No. 20230403), *Codonopsis radix* (Batch No. 20230403), *Astragali radix* (Batch No. 20230403), *Salviae miltiorrhizae radix et rhizoma* (Batch No. 20230403), *Ophiopogonis radix* (Batch No. 20230403), and *Schisandrae chinensis fructus* (Batch No. 20230403) were purchased from Guangxi Xianzhu Traditional Chinese Medicine Technology Co., Ltd. and identified by Zhang Bei, Deputy Director of the Quality Inspection Office of Liuzhou Traditional Chinese Medical Hospital; caffeic acid (Batch Number MUST‐21062110), naringin (Batch Number MUST‐20041803), luteolin (Batch Number MUST‐20102418), ononin (Batch Number MUST‐22080317), and calycosin‐7‐O‐beta‐D‐glucoside (Batch Number MUST‐23042920) were purchased from Chengdu Munster Biotechnology Co., Ltd.; baicalin (Batch Number 110715‐201821), formononetin (Batch Number 111703‐201504), rosmarinic acid (Batch Number 111871‐2007), and protocatechualdehyde (Batch Number: 110810‐200506) were purchased from China Institute for Food and Drug Control, and the purity of each compound was higher than 98.0% by HPLC; VC, H_2_O_2_, ferrous sulfate, and anhydrous ethanol (Chengdu Kelong Chemical Co., Ltd.); DPPH (Batch Number C11753367, Shanghai McLean Biochemical Technology Co., Ltd.), salicylic acid (Chengdu Kekelong Chemical Reagent Factory); formic acid (chromatographically pure, Tianjin Kemio Chemical Reagent Co., Ltd.); and acetonitrile (chromatographically pure), phosphoric acid (chromatographically pure), and methanol (chromatographically pure) were purchased from Hubei Forton Biochemical Technology Co., Ltd.; ultrapure water is prepared by GWB‐1 ultrapure water machine (Beijing Puxi General Instrument Co., Ltd.).

### 2.2 Preparation of Test Solutions

Accurately measure 10 mL of SQTM, followed by the addition of 10 mL of n‐butanol for extraction. Collect the supernatant, and add another 10 mL of n‐butanol to the filtrate for a second extraction. Combine the supernatant, evaporate to dryness, and dissolve the residue in methanol. Fix the volume to 10 mL, filter through a 0.45‐μm microporous membrane, and take the subsequent filtrate. This serves as the test solution. The test solution of *Ginseng radix et rhizoma rubra*, *Codonopsis radix*, *Astragali radix*, *Salviae miltiorrhizae radix et rhizoma*, *Ophiopogonis radix*, and *Schisandrae chinensis fructus* was obtained by the same method.

### 2.3 Preparation of Standard Solutions

Precisely weigh an appropriate amount of reference standards and dissolve them in methanol to prepare a mixed reference solution containing 40.880 μg/mL of protocatechualdehyde, 39.160 μg/mL of caffeic acid, 120.840 μg/mL of calycosin‐7‐O‐beta‐D‐glucoside, 65.160 μg/mL of naringin, 126.672 μg/mL of rosmarinic acid, 56.400 μg/mL of formononetin, 9.720 μg/mL of baicalin, 36.096 μg/mL of luteolin, and 7.305 μg/mL of ononin. Filter the solution through a 0.22‐μm membrane and collect the filtrate to obtain the reference standard solution. A series of mixed standard solutions with appropriate concentrations were prepared for analysis.

### 2.4 HPLC Chromatographic Conditions

Chromatographic separation was performed on the Agilent 1260 Infinity II HPLC system using an Agilent ZORBAX SB‐C18 chromatographic column (250 mm × 4.6 mm, 5 μm). The mobile phase consisted of water with 0.1% formic acid (A) and acetonitrile solution (B), and the gradient elution program was as follows: 0–25 min, 10%–25% B, and 25–40 min, 25%–65% B. The column temperature was maintained at 30°C, and the injection volume was 10 μL. Considering the effects of separation and the stability of system pressure, the flow rate was set at 1.0 mL/min. To ensure the simultaneous detection of all characteristic components, each exhibiting strong absorption, the detection wavelength was measured at 267 nm.

### 2.5 Fingerprint and Similarity Evaluation

Take 17 different batches of SQTM to prepare the test solution; inject samples for analysis according to chromatographic conditions. Chromatograms were recorded separately for each batch and imported into the Similarity Evaluation System for Chromatographic Fingerprints of Traditional Chinese Medicine for analysis. The average method was employed, with a time width set at 0.10 min, to generate the chromatographic fingerprint of SQTM.

### 2.6 Quantitative Analysis

Precisely pipette a series of mixed reference solutions, inject samples for measurement according to chromatographic conditions, and record the peak areas for QAMS. The relative correction factor (RCF) and relative retention time (RRT) were recorded and evaluated using different instruments, chromatographic columns, column temperatures, and flow rate conditions.

### 2.7 Verification of HPLC Method

#### 2.7.1 Verification of Fingerprint Method

Precision, stability, and repeatability tests are used to ensure the reliability and consistency of fingerprint. To verify the precision, six consecutive injections of the test solution from the same batch of SQTM were performed. To verify the repeatability, six parallel preparations of the test solution from the same batch of SQTM were made. For the stability evaluation, the SQTM test solution was injected and analyzed after 0, 2, 4, 8, 12, and 24 h. The relative standard deviation (RSD) of the RRT and relative peak area (RPA) of each common peak were calculated to verify the methodology of the fingerprint.

#### 2.7.2 Verification of Quantitative Method

To verify specificity, comparisons were made among the mixed reference solution, test solution, and negative reference solution. Standard curves of nine components were plotted with mass concentration (X) against chromatographic peak area (Y). The precision, reproducibility, and stability were verified by calculating the RSD of the retention time and content of the nine components. The precision was verified by taking the mixed reference solution for six consecutive injections. The repeatability and stability verification methods were the same as those described in Section 2.7.1. For the recovery test, six samples of SQTM with known concentrations were taken, and the reference substances of nine components were added based on a nearly 1:1 ratio. The average recovery rates and their RSD of the nine components were calculated to evaluate the accuracy of the quantitative method.

### 2.8 Measurement of Antioxidant Activity

#### 2.8.1 Preparation of Compound Samples

Seventeen batches of SQTM sample ethanol solutions with different concentrations (10, 8, 6, 4, 2, and 1 mg/mL) were prepared for subsequent use. Take the monomer reference standard of the active ingredient and prepare it into solutions at a concentration of 1.0 mg/mL using ethanol as the solvent.

#### 2.8.2 Determination of DPPH Free Radical Scavenging Rate

Prepare a DPPH solution with a concentration of 0.1 mol/L using ethanol. Aspirate 2.0 mL of the test solution into a centrifuge tube, and subsequently add 2.0 mL of the DPPH solution. The mixture was thoroughly shaken and allowed to react in the dark for 30 min [13]. Vitamin C (VC) was used as a positive control, and the absorbance value was measured at a wavelength of 517 nm. The calculation formula is as follows:

$$\text{DPPH free radical scavenging rate}\;\left(\%\right)=\left[1-\frac{\left(A1-A2\right)}{A3}\right]\times 100,$$

where *A*1 represents the absorbance value of the sample, *A*2 represents the absorbance value when ethanol replaces the sample, and *A*3 represents the absorbance value when ethanol replaces the DPPH solution. A fitting equation was obtained after fitting with the sample concentration as the abscissa *X* and the free radical scavenging rate as the ordinate *Y*. Based on this equation, the relative half maximal inhibitory concentration (IC_50_) was calculated.

#### 2.8.3 Determination of Hydroxyl Radical Scavenging Rate

Two milliliters of the test solution was aspirated into a centrifuge tube, followed by the addition of 2.0 mL of 9.0 mmol/L FeSO_4_ solution, 9.0 mmol/L salicylic acid, and 8.8 mmol/L H₂O₂. The mixture was then heated and reacted in a 37°C water bath for 30 min [14]. VC was used as a positive control, and the absorbance value Ai of the sample solution was measured at a wavelength of 510 nm. The calculation formula is as follows:

$$\text{Hydroxyl radical scavenging rate}\;\left(\%\right)=\left[1-\frac{\left(\mathrm{Ai}-\mathrm{Aio}\right)}{\mathrm{Ao}}\right]\times 100,$$

where *Aio* represents the absorbance value when distilled water replaces H₂O₂ and *Ao* represents the absorbance value when ethanol replaces the sample. A fitting equation was obtained by plotting the sample concentration as the horizontal coordinate *X* and the radical scavenging rate as the vertical coordinate *Y*. Based on this equation, the IC_50_ was then calculated.

### 2.9 Analysis of Spectrum‐Effect Relationship

#### 2.9.1 GRA

The data were imported into SPSS 26.0 for GRA with DPPH and the hydroxyl radical (OH) scavenging activity index (IC_50_) as the parent sequence and the contents of nine components in SQTM as the child sequence. The original data were first normalized using the mean value method to get dimensionless. Subsequently, the absolute difference and gray relational coefficient between each child sequence and the parent sequence were calculated. Finally, the degree of gray relation (*r*) was determined.

#### 2.9.2 BCA

The Shapiro–Wilk test was employed to assess whether the variables conform to a normal distribution, particularly in the context of small sample sizes. For variables that do not adhere to a normal distribution, Spearman rank correlation coefficients were utilized. Conversely, when the data follow a normal distribution, Pearson correlation coefficients were applied. The statistical software SPSS 26.0 was used to evaluate the correlations among the contents of nine components in SQTM and their corresponding DPPH and the hydroxyl radical scavenging activity index (IC50).

#### 2.9.3 PLSR

The contents of nine components of the samples were used as independent variables, whereas DPPH and the hydroxyl radical scavenging activity index (IC_50_) were employed as dependent variables. These variables were imported into SIMCA 14.0 for PLSR.

### 2.10 Molecular Docking Prediction

The SDF structure of ligand compounds was obtained from the PubChem database, and crystal structures of six antioxidant‐related receptors were retrieved from relevant literature and the RCSB protein database [15]. The proteins underwent routine processing, such as dehydration and charging, and were then exported in PDB format to complete the receptor preparation. The receptors and compounds were imported into AutoDock tools software to determine the position and size of the docking box and subsequently exported in PDBQT format. Following this, docking simulations between the ligand compounds and receptors were performed using AutoDock Vina software, and the results obtained were visualized and analyzed using PyMOL software.

### 2.11 Activity Verification

Take the monomer component test solution to react with the DPPH and OH working solutions, with VC serving as the positive control, and calculate the scavenging rate of each component. The experiment was repeated three times, and the mean value and standard deviation were calculated. Single‐factor analysis of variance (ANOVA) was conducted using SPSS 26.0 with a significance level of *p* < 0.05. Additionally, multiple comparisons were performed using the LSD and Duncan's methods.

## 3 Results and Discussion

### 3.1 Optimization of HPLC Chromatographic Condition

In the preliminary experiment, different extraction solvents (chloroform, ethyl acetate, and n‐butanol) were first investigated. It was found that n‐butanol worked best, showing the most abundant chromatographic peaks. Subsequently, the separation effects of different mobile phases (methanol–water, acetonitrile‐water, acetonitrile‐0.1% phosphoric acid water, and acetonitrile‐0.1% formic acid water) were examined, and the acetonitrile‐0.1% formic acid in water system exhibited the best separation performance. Furthermore, full wavelength scanning in the range of 200–400 nm using a DAD revealed that a wavelength of 267 nm could well reflect the integrity of the chromatogram. Additionally, the durability of varying flow rates (0.8, 1.0, and 1.2 mL/min) was investigated. At a flow rate of 1.0 mL/min, the number of chromatographic peaks remained stable, demonstrating better applicability and reproducibility (Figures S1–S4).

### 3.2 Methodology Verification

#### 3.2.1 Verification of Fingerprint Methodology

Using calycosin‐7‐O‐beta‐D‐glucoside, which exhibited good separation, as the reference peak, the RSDs of the precision, repeatability, and stability of the fingerprint method were all found to be below 2.47%, indicating the reliability of the method. The results of the method verification are shown in Table 1.

**TABLE 1:** The precision, repeatability, and stability of the common peaks in SQTM (*n* = 6).

Peak	Precision		Repeatability		Stability	
	RRT	RPA	RRT	RPA	RRT	RPA
1	0.09	1.56	0.08	1.64	0.18	1.81
2	0.07	2.47	0.08	2.26	0.17	2.09
4	0.08	1.42	0.06	2.22	0.22	2.17
5	0.10	1.56	0.07	2.31	0.16	1.52
6	0.10	1.46	0.09	2.33	0.18	1.36
7	0.09	1.63	0.07	2.34	0.21	1.63
8	0.10	1.57	0.10	2.01	0.25	1.39
9	0.10	2.15	0.10	2.47	0.25	2.39

#### 3.2.2 Verification of Quantitative Methodology

**FIGURE 1:**
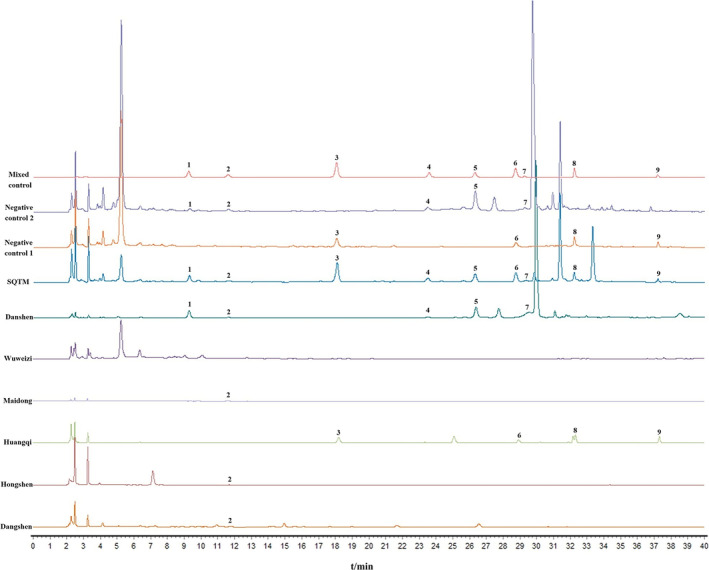
Specific analysis of HPLC chromatogram of SQTM. Negative control 1 is characterized by the absence of *Salviae miltiorrhizae radix et rhizome*, *Ginseng radix et rhizoma rubra*, *Codonopsis radix*, and *Ophiopogonis radix*. Negative control 2 lacks *Astragali radix*. Danshen symbolizes *Salviae miltiorrhizae radix et rhizome*. Wuweizi symbolizes *Schisandrae chinensis fructus*. Maidong symbolizes *Ophiopogonis radix*. Huangqi symbolizes *Astragali radix.* Hongshen symbolizes *Ginseng radix et rhizoma rubra.* Dangshen symbolizes *Codonopsis radix.* Numbers 1–9 indicate the nine common peaks for identification: (1) protocatechualdehyde, (2) caffeic acid, (3) calycosin‐7‐O‐beta‐D‐glucoside, (4) naringin, (5) rosmarinic acid, (6) ononin, (7) baicalin, (8) luteolin, and (9) formononetin.

The negative control solution did not interfere with the detection of the target components. The retention time of each component was consistent, and the chromatographic peaks exhibited good separation, showing good specificity (Figure 1). A standard curve was plotted with mass concentration (*X*) versus chromatographic peak area (*Y*), and linear regression was performed for nine components. The *R* ^2^ values of each component were all > 0.999, indicating that the nine components exhibited a good linear relationship within their respective linear ranges. Then, limits of detection (LOD) and limits of quantification (LOQ) were determined, and the results are presented in Table 2.

**TABLE 2:** Standard curves of nine kinds of standard substance (*n* = 6).

Components	Regression equation	*R* ^2^	LOD/(μg/mL)	LOQ/(μg/mL)	Linear range/(μg/mL)
Protocatechualdehyde	*Y* = 27.244*X* + 14.763	0.999 5	0.377	1.256	2.044–40.880
Caffeic acid	*Y* = 14.981*X* + 1.844	0.999 7	0.426	1.149	1.958–39.160
Calycosin‐7‐O‐beta‐D‐glucoside	*Y* = 26.368*X* + 5.281	0.999 9	0.395	1.318	6.042–120.840
Naringin	*Y* = 15.354*X* + 2.378	0.999 9	0.478	1.592	3.258–65.160
Rosmarinic acid	*Y* = 7.341 6*X* + 1.067	1.000 0	0.365	1.218	6.334–174.174
Ononin	*Y* = 30.626*X* + 7.405	0.999 9	0.633	2.110	2.820–56.400
Baicalin	*Y* = 21.596*X* + 2.548 9	0.999 3	0.101	0.338	0.486–9.720
Luteolin	*Y* = 28.249*X* + 0.564	1.000 0	0.342	1.141	1.805–36.096
Formononetin	*Y* = 39.257*X* + 1.729 9	0.999 9	0.098	0.327	0.365–7.305

The reference standard was injected for six consecutive times, and the measurement results showed that the RSD for the content of each component ranged from 1.00% to 2.35%, whereas the RSD for the retention time ranged from 0.03% to 0.45% (Table S1), demonstrating good precision of the instrument. The measurement results of six parallel test solutions showed that the RSD for the content of each component ranged from 1.56% to 2.45% and the RSD for the retention time ranged from 0.02% to 0.16% (Table S1), indicating good repeatability of the method. The test samples were injected and measured after 0, 2, 4, 8, 12, and 24 h. The results showed that the RSD for the content of each component ranged from 1.04% to 2.46% and the RSD for the retention time ranged from 0.03% to 0.47% (Table S1), manifesting good stability of the test solution. Six test samples of SQTM with known concentration were used, and the reference standards of the nine components were added in quantities close to a 1:1 ratio with the sample amounts. The measurement results showed that the average recovery rates for the components ranged from 97.48% to 103.08%, with an RSD ranging from 0.62% to 2.49% (Table S1). This indicated good accuracy of the method.

### 3.3 Establishment and Similarity Evaluation of Fingerprint

**FIGURE 2:**
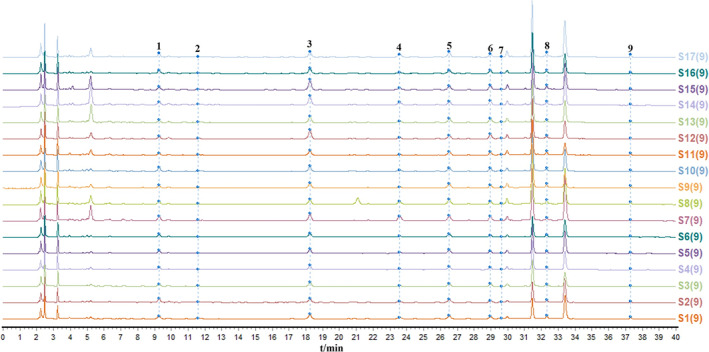
Overlapping HPLC chromatographic fingerprints of SQTM samples

After conducting multipoint correction and marker peak matching on the fingerprints of 17 batches of SQTM, with S17 serving as the control fingerprint, the results were presented in Figure 2. Among them, Peak No. 7, representing calycosin‐7‐O‐beta‐D‐glucoside, exhibited good separation and a median retention time and was selected as the reference peak. The similarity between each batch was calculated, and all batches exhibited a similarity of above 0.950, indicating the stability of the chemical composition among the samples of various batches.

### 3.4 Analysis of QAMS Methodology

#### 3.4.1 Calculation of Relative Correction Factor (RCF)

The RCFs of protocatechualdehyde, caffeic acid, naringin, rosmarinic acid, ononin, baicalin, luteolin, and formononetin were calculated with calycosin‐7‐O‐beta‐D‐glucoside as the internal standard.

The formula is as follows:

$$f_{i}=\frac{\left(A_{s}\times C_{i}\right)}{\left(A_{i}\times C_{s}\right)},$$

where $A_{s}$, $A_{i}$, $C_{s}$, and $C_{i}$ represent the peak area of the internal standard and the component, and the concentration of the internal standard and the component, respectively. The results of RCFs for the nine components were 0.93, 1.74, 1.67, 3.61, 0.85, 1.13, 0.95, and 0.64, and their RSDs were 0.52%, 1.73%, 1.73%, 0.38%, 0.12%, 0.08%, 1.37%, and 2.08%, respectively. The relative retention times were 0.51, 0.64, 1.31, 1.46, 1.59, 1.62, 1.79, and 2.06, and the RSDs were 0.11%, 0.05%, 0.03%, 0.02%, 0.04%, 0.04%, 0.03%, and 0.04%, respectively.

#### 3.4.2 Evaluation of RCF and RRT

With other chromatographic conditions unchanged, the effect of various factors on the relative correction factor of each component was investigated, including different instruments (SHIMDZHU LC‐40 DXR, Agilent 1260 Infinity II HPLC), chromatographic columns (InsertSustain AQ‐C18 [250 mm × 4.6 mm, 5 μm], Dikma Diamond C18 [250 mm × 4.6 mm, 5 μm], Agilent ZORBAX SB‐C18 [250 mm × 4.6 mm, 5 μm]), column temperature (25°C, 30°C, and 35°C) and flow rate (0.8, 1.0, and 1.2 mL/min). The results are depicted in Figure S5; the RSD values of all RCFs under different experimental conditions ranged from 0.05% to 0.97%, indicating that different instruments, chromatographic columns, column temperatures, and flow rates had little influence on the relative correction factors of the nine components.

With calycosin‐7‐O‐beta‐D‐glucoside as the internal standard, the investigation on the influence of different instruments, chromatographic columns, column temperatures, and flow rates on the relative retention times (RRT) of protocatechualdehyde, caffeic acid, naringin, rosmarinic acid, ononin, baicalin, luteolin, and formononetin was performed. The results are shown in Figure S6; the RSD values of all RRTs under different experimental conditions ranged from 0.07% to 0.11%, indicating that this method can be used for the accurate localization of the target chromatographic peak.

#### 3.4.3 Evaluation of Consistency Between QAMS and ESM

**FIGURE 3:**
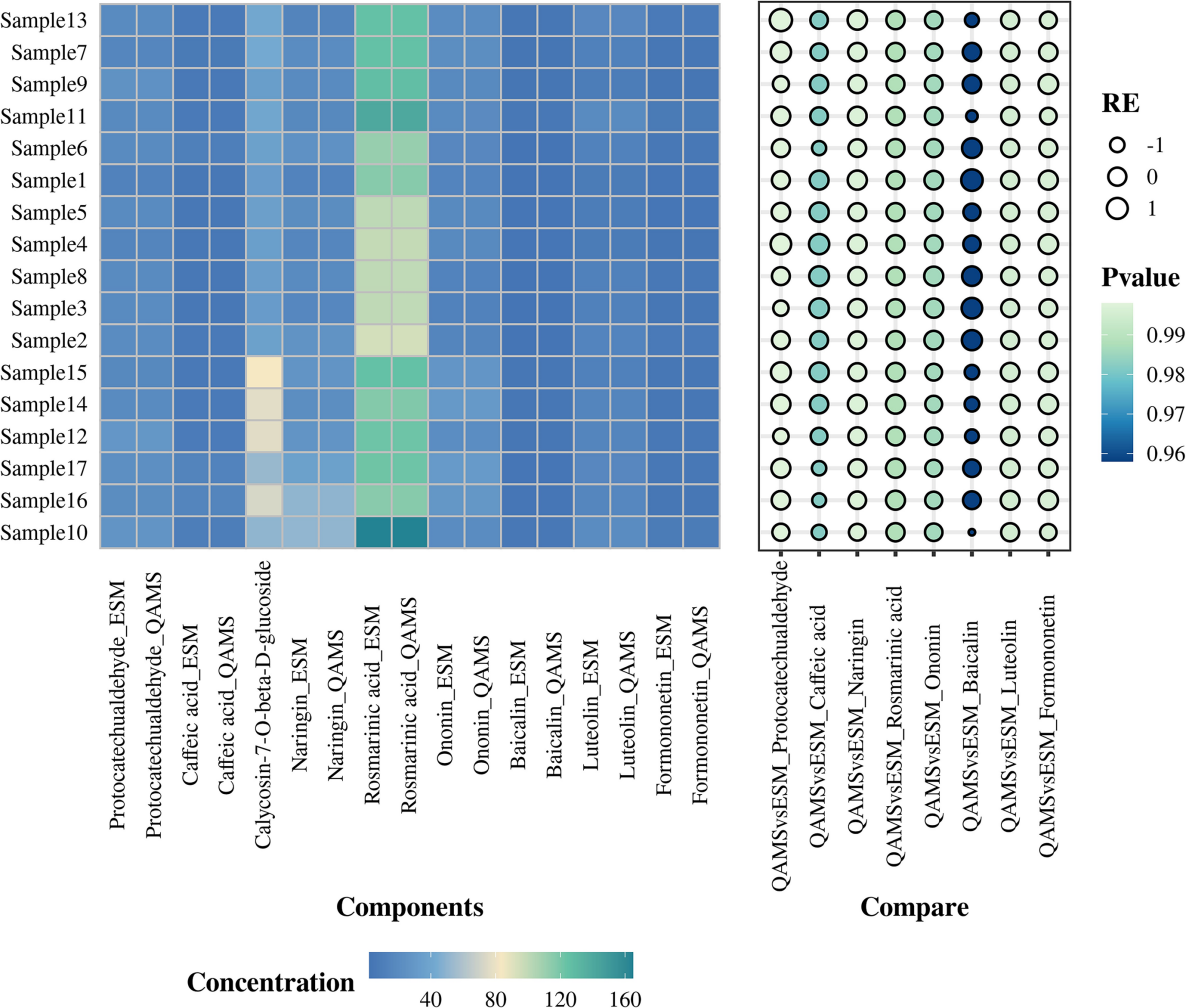
Results of evaluation of consistency between QAMS and ESM. The squares represent the concentration of the ingredients, and the greater the concentration, the greener the color. The size of the bubble indicates the size of RE, and the larger the RE, the larger the bubble; the color of the bubble indicates the size of the *p* value, and the larger the *p* value, the greener the color.

Calycosin‐7‐O‐beta‐D‐glucoside, with its stable chemical properties and good separation efficiency, was utilized as an internal standard to establish a QAMS method for the determination of eight components in SQTM. The external standard method (ESM) was used to calculate the contents of the nine components, and a further comparison of the content results for the eight components calculated by both ESM and QAMS methods was conducted (Table S2). The two detection results are relatively close, with a relative error (RE) of less than 1.85%. Additionally, the difference between the two methods was analyzed by the *t* test, and there is no statistical difference between the results measured by the QAMS and ESM methods (*p* > 0.05), as shown in Figure 3. The established method can be used for the simultaneous content determination of eight components in SQTM.

Consequently, the content determination method of the eight components based on QAMS was established. It is suggested that in the absence of reference standards, the quality control of the eight components in SQTM can be achieved by calculating their contents through RCF values and chromatographic peak positioning. As an effective evaluation method of SQTM quality, this method can provide a reference and basis for the quality evaluation of SQTM.

### 3.5 Antioxidant Test

**FIGURE 4:**
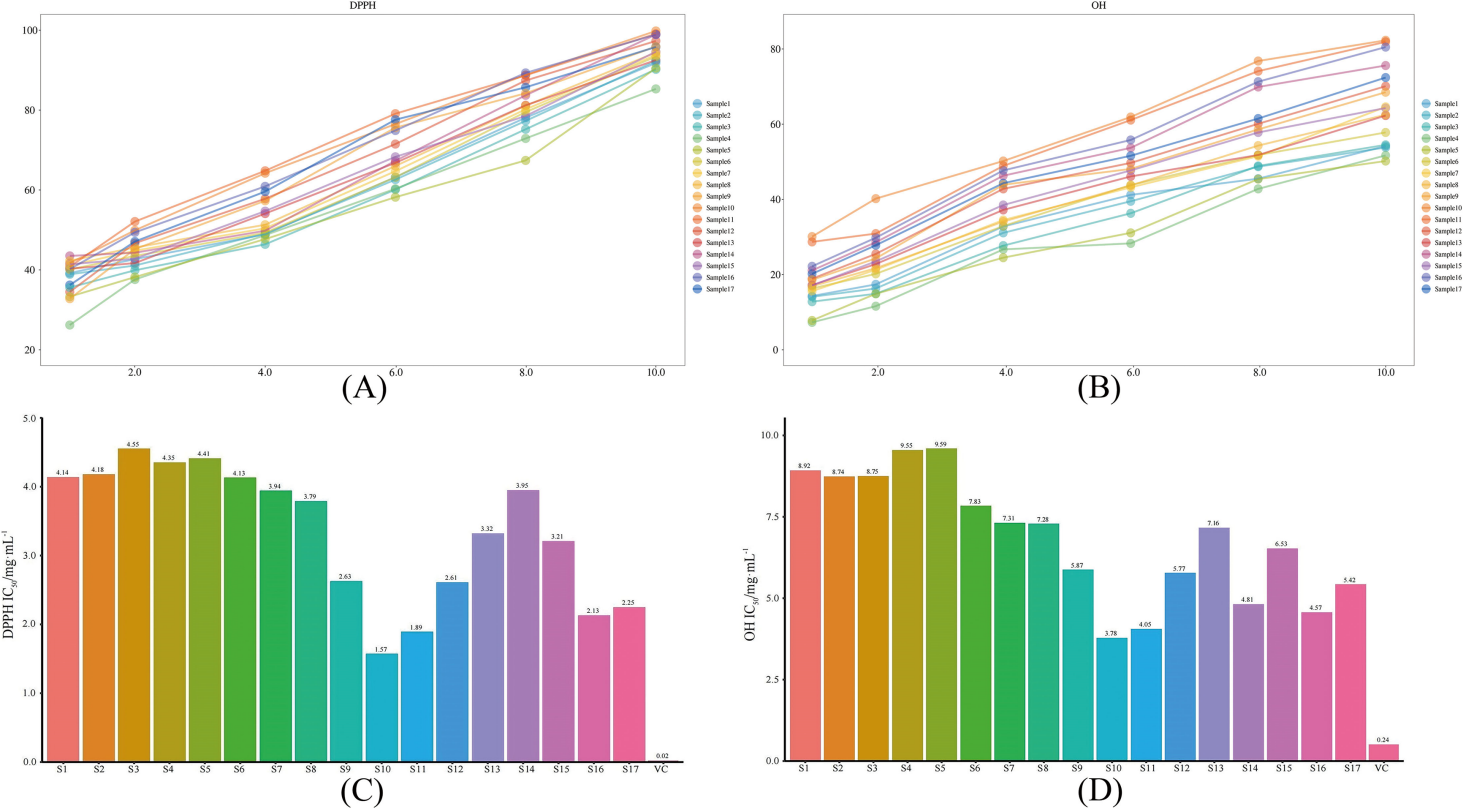
Scavenging ability of SQTM at different concentrations on DPPH (A) and OH (B) free radicals and IC50 values of 17 batches of SQTM and VC on DPPH (C) and OH (D).

The results of antioxidant test showed that 17 batches of samples exhibited certain scavenging effects on DPPH (Figure 4A) and hydroxyl free radicals (Figure 4B). Significant differences in antioxidant capacity were observed among different batches of samples. However, these effects were weaker than that of the positive control VC (Figure 4C,D). The IC_50_ values, representing the DPPH radical scavenging capacity ranged from 1.57 to 4.55 mg/mL, with the order of scavenging capacity being S10 > S11 > S16 > S17 > S12 > S9 > S15 > S13 > S8 > S7 > S14 > S6 > S1 > S2 > S4 > S5 > S3 (Figure 4C); the IC_50_ values for the hydroxyl radical scavenging capacity ranged from 3.78 to 9.59 mg/mL, with the order being S10 > S11 > S16 > S14 > S17 > S12 > S9 > S15 > S13 > S8 > S7 > S6 > S2 > S3 > S1 > S4 > S5 (Figure 4D). On the whole, the two batches of samples, S10 and S11, demonstrated relatively higher scavenging abilities for both DPPH and OH radicals. Similarly, we observed that the concentrations of rosmarinic acid and luteolin in the S10 and S11 batches were significantly higher than those found in other batches. However, no obvious difference in scavenging capacity was observed among the samples of S1–S8, S13–S15, S9, S12, S16, and S17. These phenomena indicate that the potential antioxidant active compounds, such as rosmarinic acid and luteolin, may vary between batches due to a complex interplay of various external factors, including the origin, processing, and preparation process of medicinal materials. Consequently, this variability leads to differences in antioxidant efficacy among the different batches.

### 3.6 Spectrum‐Effect Relationship

#### 3.6.1 GRA

**FIGURE 5:**
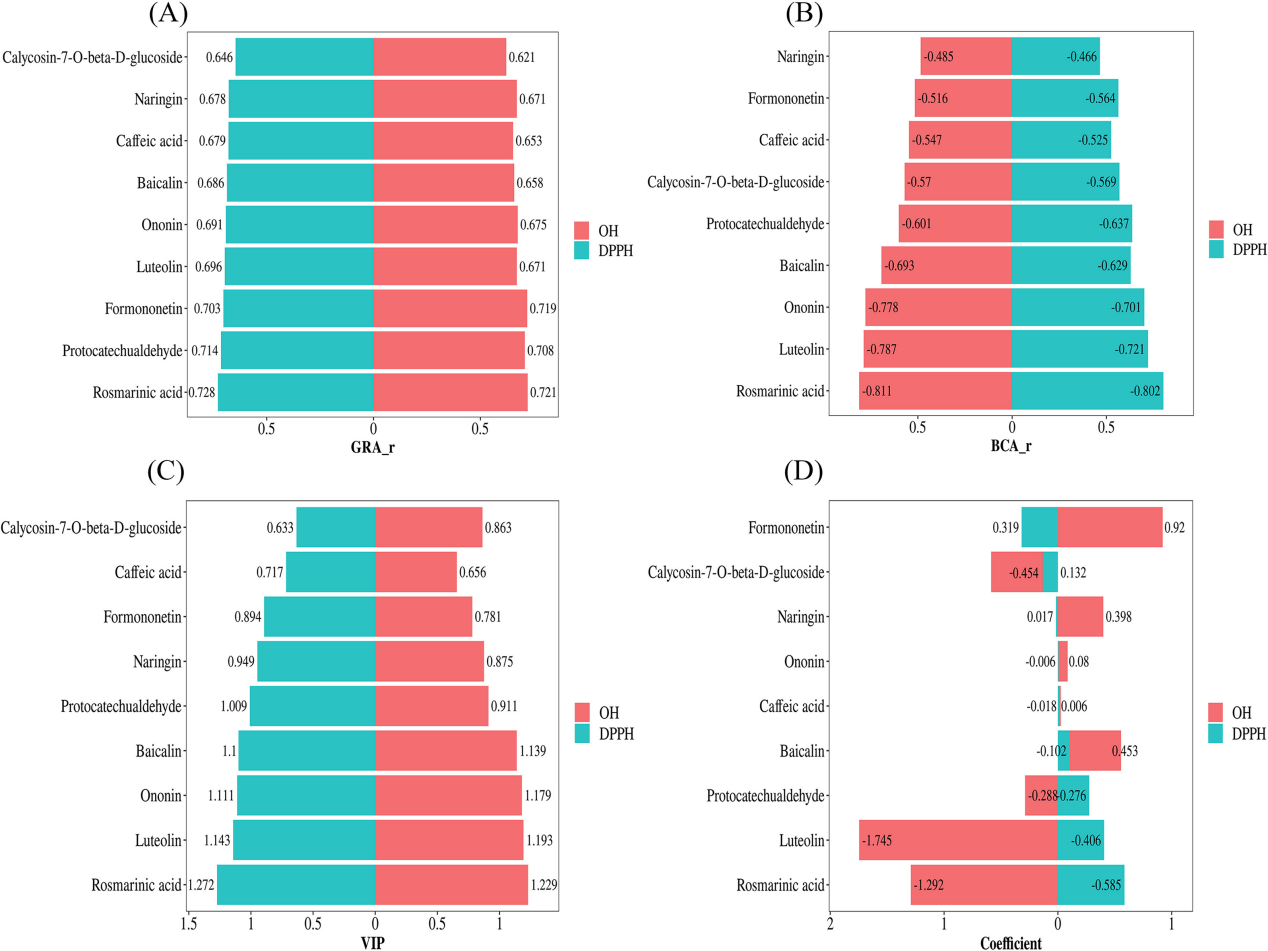
GRA results (A), BCA results (B), VIP contribution diagram (C), and standardized regression coefficient diagram (D) of nine components

It is generally accepted that when *r* > 0.5, there is a certain relationship between the subsequence and the parent sequence, and a higher value indicates a stronger correlation between the component and antioxidant activity. The *r* values of the nine components with respect to their DPPH and hydroxyl radical scavenging capacity were all greater than 0.5 (Figure 5A), indicating that each component contributed to the antioxidant effect to varying degrees. It can be seen from Figure 5A that the top five components in both GRA rankings of the two antioxidant capacities are rosmarinic acid, protocatechualdehyde, formononetin, luteolin, and ononin. However, the GRA is unable to ascertain whether these components exert a positive or negative influence on the free radical scavenging rate. Additionally, results derived from different methods and calculation formulas may yield varying outcomes. Therefore, it is essential to explore other correlations to further elucidate the relationship between these components and the DPPH and hydroxyl free radical scavenging rates.

#### 3.6.2 BCA

The correlation coefficient serves as a precise metric for quantifying the strength of linear relationships between two continuous variables. A correlation coefficient greater than 0 indicates a positive correlation, whereas a value less than 0 signifies a negative correlation. Specifically, when *r* exceeds 0.4, it suggests a strong relationship; the higher the value, the stronger the association between this component and its antioxidant activity. In our study, all nine constituents exhibited correlation coefficients for DPPH and hydroxyl radical scavenging (IC50) that were greater than 0.4, demonstrating that their antioxidant effects are interconnected. The order of correlation coefficients for DPPH free radical scavenging effect (IC50) was as follows: rosmarinic acid > luteolin > ononin > protocatechualdehyde > baicalin > calycosin‐7‐O‐beta‐D‐glucoside > formononetin > caffeic acid > naringin. For hydroxyl free radical scavenging, the sequence was rosmarinic acid > luteolin > ononin > baicalin > protocatechualdehyde > calycosin‐7‐O‐beta‐D‐glucoside > caffeic acid > formononetin > naringin; notably, these coefficients were all negative, indicating an enhancement in antioxidant scavenging capacity across all nine components (Figure 5B). Furthermore, BCA correlation results corroborated that the nine components significantly influencing DPPH and hydroxyl radical scavenging rates were largely consistent with one another. When compared to GRA results, there was alignment in identifying protocatechualdehyde, rosmarinic acid, luteolin, and ononin as key constituents.

#### 3.6.3 PLSR

Because GRA and BCA focus on the degree of correlation between components and activity, it is necessary to further integrate PLSR to comprehensively describe the contribution rates and interactions of each component to the activity indicator (IC_50_). In general, the independent variable with a VIP value greater than 0.5 is considered meaningful for explaining the dependent variable; the larger the VIP value, the greater its contribution level. When the VIP exceeds 1, this component serves as a crucial influence index. Meanwhile, when the standardized regression coefficient is positive, it indicates a positive correlation between component content and antioxidant effect; otherwise, it is a negative correlation. According to Figure 5C, all nine components in SQTM have VIP values greater than 0.5 for DPPH radical scavenging activity, suggesting that these components are closely related to DPPH radical reaction. The contribution of the nine components to DPPH radical scavenging activity is ranked as follows: rosmarinic acid > luteolin > ononin > baicalin > protocatechualdehyde > naringin > formononetin > caffeic acid > calycosin‐7‐O‐beta‐D‐glucoside. All nine components in SQTM have VIP values greater than 0.5 for hydroxyl radical scavenging activity, demonstrating that these components are closely related to hydroxyl radical reaction. The contribution of these nine components to hydroxyl radical scavenging activity is ranked as follows: rosmarinic acid > luteolin > ononin > baicalin > protocatechualdehyde > naringin > calycosin‐7‐O‐beta‐D‐glucoside > formononetin > caffeic acid. Among them, the VIP values for rosmarinic acid, luteolin, and ononin in relation to their antioxidant capacities were greater than 1. Additionally, the VIP value for protocatechualdehyde's DPPH radical scavenging activity also exceeded 1, whereas the VIP for hydroxy radical scavenging activity was measured at 0.911, close to 1. These phenomena indicate that these compounds are more significant than average in explaining variations in dependent variables. Consequently, they could be considered as potential quality markers of antioxidant activity.

Although the VIP value can assess the significance of various chemical components, it does not account for the interactions—whether promoting or inhibiting—between these components and specific indicators. Consequently, regression coefficients for different components were calculated. The DPPH regression coefficients for rosmarinic acid, luteolin, and protocatechualdehyde were −0.585, 0.406, and −0.276, respectively; meanwhile, the hydroxyl radical regression coefficients were −1.292, −1.745, and −0.288 (Figure 5D). These results indicate a significant negative correlation among these compounds, suggesting that they play a crucial role in scavenging DPPH and hydroxyl radicals. Additionally, the regression coefficients for ononin (DPPH: −0.006, hydroxyl: 0.08) were relatively small, and the effect on hydroxyl radical scavenging rate (IC_50_) exhibited a weak positive correlation. Given the complexity inherent in multivariate analysis, leading to smaller regression coefficients, suggests that there is slight inhibition of hydroxyl radical scavenging activity, however, it is noteworthy that all VIP values associated with ononin exceeded 1.0, indicating substantial significance in this context. Therefore, it is hypothesized that ononin may exert a synergistic effect alongside other constituents to collectively enhance DPPH and hydroxyl radical scavenging activities.

When comparing results from GRA and BCA correlations with those obtained through PLSR analysis, a high level of agreement was observed overall. Ultimately, based on combined findings from GRA, BCA, and PLSR analyses, rosmarinic acid, luteolin, ononin, and protocatechualdehyde have been identified as candidate markers indicative of antioxidant activity within SQTM.

### 3.7 Comprehensive Analysis of Spectrum‐Effect Relationship

Significant variations in the ability to scavenge DPPH and hydroxyl radicals were observed among different batches of SQTM samples, which are presumably attributed to comprehensive quality differences stemming from factors such as the origin, processing, and preparation procedures of the medicinal materials. The results of GRA and BCA showed that all nine components were related to antioxidant activity and all of them contributed to enhancing antioxidant scavenging ability. Notably, rosmarinic acid, luteolin, protocatechualdehyde, and ononin showed the closest relationship with antioxidant effects. In PLSR, they also have significant impacts on DPPH and hydroxyl radical scavenging activities. Furthermore, the positive and negative correlations between different components and their respective activity indicators were characterized by chemical regression coefficients. Substances exhibiting high GRA and BCA values but demonstrating inhibitory effects on antioxidant capacity were excluded from consideration, thus confirming that the three analyses are essentially complementary to each other. The chemical structures of protocatechualdehyde, rosmarinic acid, luteolin, and ononin all contain phenolic hydroxyl groups, which are crucial for their antioxidant activity. These groups enhance antioxidant activity by donating hydrogen atoms to scavenge radicals [16, 17]. Rosmarinic acid and luteolin can regulate the activity of oxidative enzymes such as superoxide dismutase (SOD), glutathione peroxidase (GSH‐Px), and catalase (CAT), or chelate transition metal ions to reduce their catalytic activity, thereby exerting antioxidant effects [18, 19, 20, 21, 22].

### 3.8 Activity Verification of Potential Antioxidant Components

#### 3.8.1 Antioxidant Targets

According to the literature, CYP2C9 and CYP3A4 belong to the cytochrome P450 (CYP450) enzyme family. Under normal physiological conditions, they maintain redox balance by catalyzing the redox reaction in the metabolic pathway. However, under pathological conditions, gene overexpression can lead to the excessive production of by‐product ROS radicals, thus exacerbating various metabolic abnormalities such as oxidative stress and inflammatory reactions [23, 24]. HO‐1 (heme oxygenase‐1) and HO‐2 (heme oxygenase‐2) are two isoenzymes of heme oxygenase (HO), whose primary function is to catalyze the degradation of heme into biliverdin, CO, and Fe^2+^. Biliverdin can scavenge ROS, whereas CO can regulate signaling pathways such as PI3K/Akt and HIF‐1 to affect the expression of antioxidant enzymes [25]. HIF‐1α can promote the expression of antioxidant enzyme genes such as superoxide dismutase (SOD) and glutathione peroxidase (GSH‐Px), thereby facilitating the removal of ROS and alleviating oxidative stress [26]. LOX‐5 can catalyze the conversion of arachidonic acid (AA) into leukotriene A4 (LTA4), leading to the production of reactive oxygen species (ROS) [27].

#### 3.8.2 Molecular Docking

**FIGURE 6:**
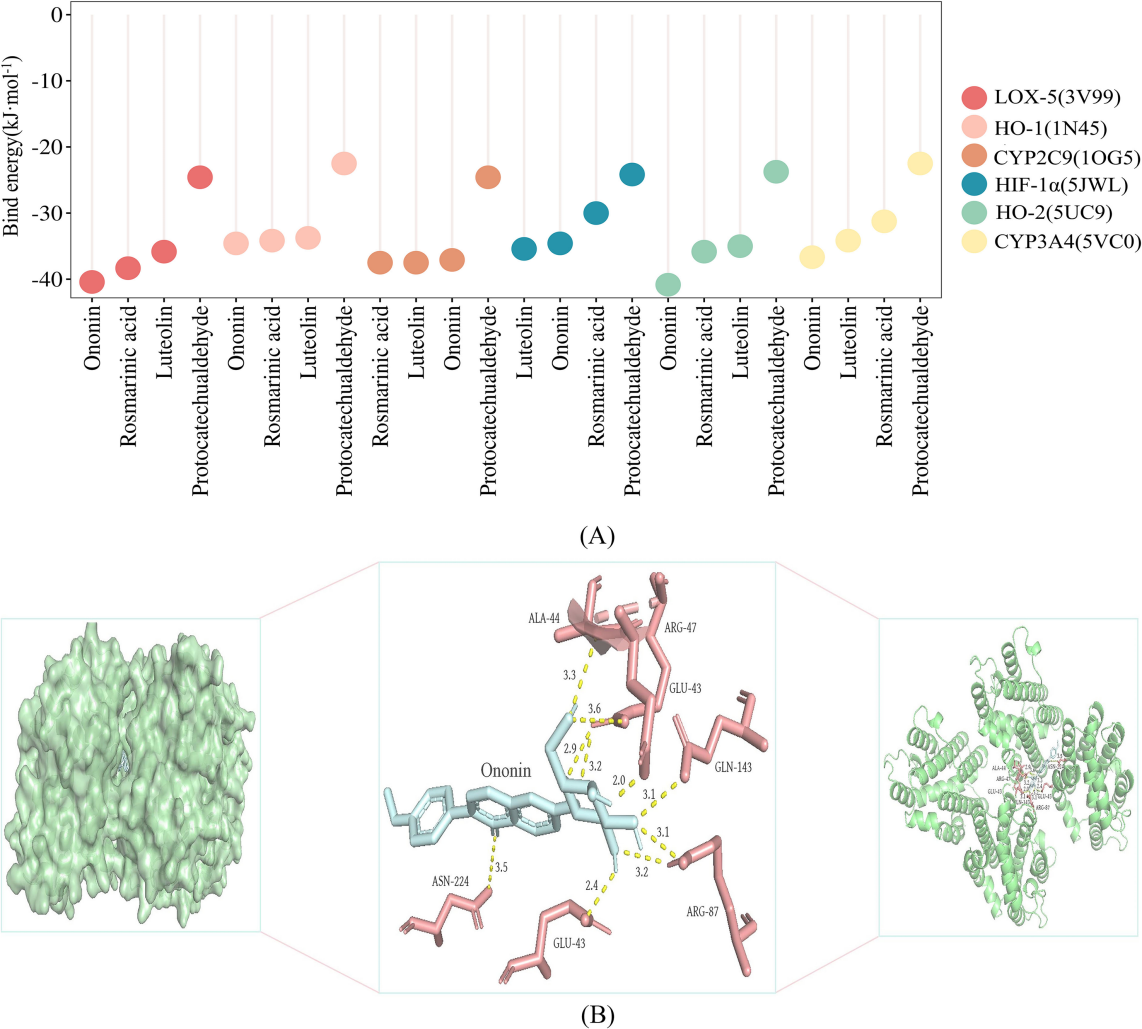
Schematic diagram of molecular docking binding energy results (A) and 3D model diagram of interaction between ononin and HO‐2 (B).

Through a comprehensive analysis of the spectrum‐effect relationship, four alternative components with different quality and antioxidant activities, namely, rosmarinic acid, luteolin, ononin, and protocatechualdehyde, were screened out, which essentially met the basic characteristics of specificity, stability, measurability, and effectiveness as quality markers. They were presumed to be the quality markers for SQTM to exert antioxidant activity. To further investigate the correlation between these four components and antioxidant activity, molecular docking was used to preliminarily predict their binding mechanisms with relevant targets. A smaller binding energy between a compound and its target indicates the release of more energy and a more stable conformation of the binding complex. It is generally accepted that a binding energy less than −5.0 kJ/mol is considered to indicate good binding activity whereas a binding energy less than −7.0 kJ/mol suggests extremely strong binding activity. According to the results in Figure 6A, it can be observed that the four active components exhibit good affinity with multiple antioxidant receptor proteins. Among them, ononin demonstrates the best binding capacity with HO‐2, and a detailed docking schematic is illustrated in Figure 6B. The strong binding force between the molecule and its target is ensured through interaction with amino acid residues. Specifically, ononin binds to HO‐2 and forms bonds with residues such as ARG‐47, ARG‐87, GLU‐43, ASN‐224, GLN‐143, and ALA‐44, with an average bond length of 3.0 Å. The four highly active components may exert their antioxidant effects by participating in the enzymatic oxidation system to regulate oxidative stress.

#### 3.8.3 Antioxidant Activity

**FIGURE 7:**
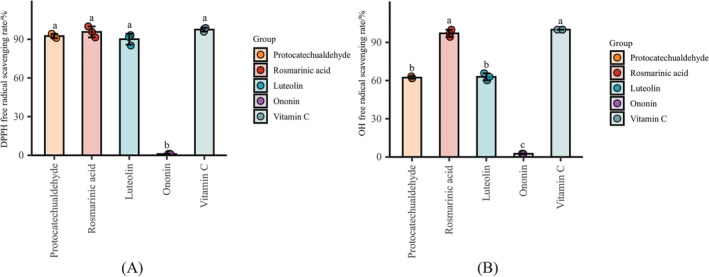
DPPH (A) and hydroxyl (B) radical scavenging rate (%) of monomer compounds. Different letters indicate significant differences (*p* < 0.05), and the same letters indicate no significant differences (*p* > 0.05).

The antioxidant results of monomer active components are shown in Figure 7. The results indicated that the four active components exhibited certain scavenging activities on DPPH and hydroxyl radicals. Among them, rosmarinic acid demonstrated the strongest scavenging activity on DPPH and hydroxyl radicals, whereas ononin exhibited the weakest. Consequently, protocatechualdehyde, rosmarinic acid, and luteolin were finally proposed as quality markers for the antioxidant activity of SQTM.

### 3.9 Comprehensive Analysis of Quality Control Indicators

Based on the results of spectrum‐effect screening, further molecular docking predictions were conducted on potential monomer components. It was found that rosmarinic acid, luteolin, ononin, and protocatechualdehyde could all bind to various antioxidant‐related targets with good binding ability. The subsequent in vitro antioxidant activity experiments demonstrated that rosmarinic acid exhibited a high scavenging capacity towards both types of free radicals, followed by luteolin and protocatechualdehyde, and ononin showed the weakest DPPH and hydroxyl scavenging activities. DPPH and hydroxyl radicals terminate free radical reactions through electron transfer and hydrogen transfer, respectively, with phenol hydroxyl groups providing dissociable protons or electrons for scavenging. Ononin belongs to the isoflavone class of compounds, and due to its relatively low number of phenolic hydroxyl groups and the instability of the cationic radical structure formed after electron transfer, its DPPH and hydroxyl scavenging activities are relatively weak.

In conclusion, rosmarinic acid, luteolin, and protocatechualdehyde may exert antioxidant effects by scavenging DPPH and hydroxyl free radicals and binding to multiple core targets. It is suggested that they be considered as quality markers for the antioxidant effect of SQTM. Meanwhile, studies have revealed that luteolin can inhibit the proliferation and migration of VSMCs by suppressing TGFBR1 signaling, regulate NOX4 to attenuate TNF‐α‐induced oxidative stress and antiatherosclerosis, and reduce inflammation and oxidative damage through the activation of Tristelaprolin (TTP) associated with IL‐6 mRNA stability and the NOX4/ROS‐NF‐κB pathway [28, 29]. Protocatechualdehyde can increase pericyte coverage through the TGF‐β1/TGFBR1/Smad2/3 signaling, thereby blocking ox‐LDL‐induced damage and improving the stability of atherosclerotic plaques [30]. Rosmarinic acid enhances antioxidant capacity and improves cellular and vascular calcification by regulating the Nrf2 pathway; it upregulates the expression of Bcl‐2, HO‐1, Nrf2, and SOD and significantly downregulates the expression of Bax and MDA, thereby protecting the brain from acute ischemic injury [31, 32]. Literature research has shown that rosmarinic acid, luteolin, and protocatechualdehyde exhibit protective effects on the cardiovascular system and may be the primary material basis for the medicinal effect of SQTM, further supporting their use as quality markers for evaluating the quality of SQTM. In addition, through an analysis of the relationship between content and activity, this paper found a positive correlation between rosmarinic acid, luteolin, protocatechualdehyde, and the antioxidant effect of SQTM. The molecular docking and literature research also showed that these three compounds may mediate multiple related targets to regulate the oxidative stress system, implying that the antioxidant effect of SQTM may be a joint result of different interaction effects among its constituents. Therefore, in subsequent research, a deeper exploration of the interaction relationships among these components can be conducted to investigate the antioxidant action mechanism of SQTM.

In summary, this study identified nine characteristic components by analyzing the SQTM fingerprint of 17 batches, and the content of eight components was determined using QAMS. Notably, the RE% of eight components between QAMS and ESM is less than 5%, indicating that QAMS can be effectively applied in the evaluation of SQTM. The scavenging ability of DPPH and hydroxyl radicals in 17 batches of SQTM was determined. It was found that there was a great difference in antioxidant ability among different batches. Combined with the spectrum‐effect relationship, it was assumed that the difference might be related to the content variations of candidate markers of antioxidant activity, rosmarinic acid, luteolin, protocatechualdehyde, and ononin. Rosmarinic acid, luteolin, and protocatechualdehyde all showed strong antioxidant activity in molecular docking and monomer component antioxidant activity test, which may be the main pharmacodynamic substance foundation of SQTM and may be used as the Q‐Markers of SQTM antioxidant effect. This study correlates the fingerprint of SQTM with its antioxidant effect for the first time and further conducts a correlation analysis between the spectrum of chemical information and the efficacy of pharmacological activity. It provides a reference for the subsequent quality evaluation, control, and the establishment of quality standardization, promoting the long‐term development in the quality control of SQTM. It offers a new idea for evaluating the quality indicators of SQTM, the pharmacological basis, and action mechanism of antioxidation.

## Supporting information


**Figure S1** HPLC chromatograms of SQTM samples under three different extraction solutions.
**Figure S2** HPLC chromatograms of SQTM samples in four different mobile phase systems.
**Figure S3** HPLC chromatograms of SQTM samples at eight different wavelengths.
**Figure S4** HPLC chromatograms of SQTM samples at three different flow rates.
**Table S1** Method validation for the simultaneous quantification of the nine constituents in SQTM (*n* = 6)
**Figure S5** Influence of different instruments, chromatographic columns, column temperature and flow rates on RCFs. The size of the bubble corresponds to the magnitude of the fi value; a larger bubble indicates a higher fi value. The color of the bubble reflects the RSD value, with a bluer hue signifying a greater RSD.
**Figure S6** Effect of different instruments, chromatographic columns, column temperatures and flow rates on RRTs. The size of the bubble corresponds to the magnitude of the RRT value; a larger bubble indicates a higher RRT value. The color of the bubble reflects the RSD value, with a bluer hue signifying a greater RSD.
**Table S2** Contents determination results of nine ingredients by QAMS and ESM (μg/mL)

## Data Availability

The data that support the findings of this study are available in the supporting information of this article. Further data are available from the corresponding author upon reasonable request.

## References

[1] G. A. Roth , G. A. Mensah , and V. Fuster , “The Global Burden of Cardiovascular Diseases and Risks: A Compass for Global Action,” Journal of the American College of Cardiology 76, no. 25 (2020): 2980–2981, 10.1016/j.jacc.2020.11.021. 33309174

[2] Y. Li , G. Y. Cao , W. Z. Jing , et al., “Global Trends and Regional Differences in Incidence and Mortality of Cardiovascular Disease, 1990–2019: Findings From 2019 Global Burden of Disease Study,” European Journal of Preventive Cardiology 30, no. 3 (2023): 276–286, 10.1093/eurjpc/zwac285. 36458973

[3] X. Yang , Y. Yang , J. Guo , et al., “Targeting the Epigenome in In‐Stent Restenosis: From Mechanisms to Therapy,” Molecular Therapy ‐ Nucleic Acids 23 (2021): 1136–1160, 10.1016/j.omtn.2021.01.024. 33664994 PMC7896131

[4] D. Wang , X. Gu , K. Fang , B. Fu , Y. Liu , and X. Di , “Study on Quality Control of Zuojin Pill by HPLC Fingerprint With Quantitative Analysis of Multi‐Components by Single Marker Method and Antioxidant Activity Analysis,” Journal of Pharmaceutical and Biomedical Analysis 225 (2023): 115075, 10.1016/j.jpba.2022.115075. 36603393

[5] X. Lu , Y. Jin , Y. Wang , Y. Chen , and X. Fan , “Multimodal Integrated Strategy for the Discovery and Identification of Quality Markers in Traditional Chinese Medicine,” Journal of Pharmaceutical Analysis 12, no. 5 (2022): 701–710, 10.1016/j.jpha.2022.05.001. 36320607 PMC9615540

[6] Q. LuoRong , L. H. Tan , B. Yu , et al., “Comprehensive Quality Evaluation of Lysimachia Christinae Hance via Fingerprint, Spectrum‐Effect Relationship, and Quantitative Analyses of Multiple Components by Single Marker,” Phytochemical Analysis 35, no. 6 (2024): 1527–1536, 10.1002/pca.3394. 38772567

[7] J. Zhang , X. Yu , R. Yang , B. Zheng , Y. Zhang , and F. Zhang , “Quality Evaluation of Lonicerae Japonicae Flos From Different Origins Based on High‐Performance Liquid Chromatography (HPLC) Fingerprinting and Multicomponent Quantitative Analysis Combined With Chemical Pattern Recognition,” Phytochemical Analysis 35, no. 4 (2024): 647–663, 10.1002/pca.3319. 38185766

[8] Q. Wang , G. Chen , X. Chen , et al., “Development of a Three‐Step‐Based Novel Strategy Integrating DMPK With Network Pharmacology and Bioactivity Evaluation for the Discovery of Q‐Markers of Traditional Chinese Medicine Prescriptions: Danlou Tablet as an Example,” Phytomedicine 108 (2023): 154511, 10.1016/j.phymed.2022.154511. 36334388

[9] X. Shan , X. Yang , D. Li , et al., “Research on the Quality Markers of Antioxidant Activity of Kai‐Xin‐San Based on the Spectrum‐Effect Relationship,” Frontiers in Pharmacology 14 (2023): 1270836, 10.3389/fphar.2023.1270836. 38205371 PMC10777484

[10] H. Li , H. Zhao , L. Chen , et al., “Spectrum‐Effect Relationship Between HPLC Fingerprints and Antioxidant Activity of Qi‐Fu‐Yin Based on Multiple Statistical Correlation Analysis,” Phytochemical Analysis 35, no. 7 (2024): 1565–1576, 10.1002/pca.3396. 38777368

[11] Y. Zhang , W. W. Li , Y. Wang , et al., “Investigation of the Material Basis and Mechanism of Lizhong Decoction in Ameliorating Ulcerative Colitis Based on Spectrum‐Effect Relationship and Network Pharmacology,” Journal of Ethnopharmacology 323 (2024): 117666, 10.1016/j.jep.2023.117666. 38159822

[12] Q. Yan , S. Liu , Y. Sun , et al., “Targeting Oxidative Stress as a Preventive and Therapeutic Approach for Cardiovascular Disease,” Journal of Translational Medicine 21, no. 1 (2023): 519, 10.1186/s12967-023-04361-7. 37533007 PMC10394930

[13] S. Zhao , Z. Han , L. Yang , B. Hong , and H. Zhu , “Extraction, Characterization and Antioxidant Activity Evaluation of Polysaccharides From *Smilacina japonica* ,” International Journal of Biological Macromolecules 151 (2020): 576–583, 10.1016/j.ijbiomac.2020.02.015. 32061692

[14] S. Wang , G. Li , X. Zhang , et al., “Structural Characterization and Antioxidant Activity of *Polygonatum sibiricum* Polysaccharides,” Carbohydrate Polymers 291 (2022): 119524, 10.1016/j.carbpol.2022.119524. 35698327

[15] P. Minchán‐Herrera , R. O. Ybañez‐Julca , I. M. Quispe‐Díaz , et al., “Valeriana pilosa Roots Essential Oil: Chemical Composition, Antioxidant Activities, and Molecular Docking Studies on Enzymes Involved in Redox Biological Processes,” Antioxidants (Basel) 11, no. 7 (2022): 1337, 10.3390/antiox11071337. 35883828 PMC9311991

[16] A. Gasmi , P. K. Mujawdiya , S. Noor , et al., “Polyphenols in Metabolic Diseases,” Molecules 27, no. 19 (2022): 6280, 10.3390/molecules2719628. 36234817 PMC9570923

[17] N. Żurek , A. Pawłowska , K. Pycia , D. Grabek‐Lejko , and I. T. Kapusta , “Phenolic Profile and Antioxidant, Antibacterial, and Antiproliferative Activity of Juglans Regia L. Male Flowers,” Molecules 27, no. 9 (2022): 2762, 10.3390/molecules270927620. 35566113 PMC9101975

[18] A. Dahchour , “Anxiolytic and Antidepressive Potentials of Rosmarinic Acid: A Review With a Focus on Antioxidant and Anti‐Inflammatory Effects,” Pharmacological Research 184 (2022): 106421, 10.1016/j.phrs.2022.106421. 36096427

[19] S. Noor , T. Mohammad , M. A. Rub , et al., “Biomedical Features and Therapeutic Potential of Rosmarinic Acid,” Archives of Pharmacal Research 45, no. 4 (2022): 205–228, 10.1007/s12272-022-01378-2. 35391712 PMC8989115

[20] M. D. S. S. Chagas , M. D. Behrens , C. J. Moragas‐Tellis , G. X. M. Penedo , A. R. Silva , and C. F. Gonçalves‐de‐Albuquerque , “Flavonols and Flavones as Potential Anti‐Inflammatory, Antioxidant, and Antibacterial Compounds,” Oxidative Medicine and Cellular Longevity 2022 (2022): 9966750, 10.1155/2022/9966750. 36111166 PMC9470311

[21] D. K. Sahoo , R. M. Heilmann , B. Paital , et al., “Oxidative Stress, Hormones, and Effects of Natural Antioxidants on Intestinal Inflammation in Inflammatory Bowel Disease,” Frontiers in Endocrinology (Lausanne) 14 (2023): 1217165, 10.3389/fendo.2023.1217165. PMC10493311 37701897

[22] S. M. Ahmadi , R. Farhoosh , A. Sharif , and M. Rezaie , “Structure‐Antioxidant Activity Relationships of Luteolin and Catechin,” Journal of Food Science 85, no. 2 (2020): 298–305, 10.1111/1750-3841.14994. 31957877

[23] D. R. Flora , A. E. Rettie , R. C. Brundage , and T. S. Tracy , “CYP2C9 Genotype‐Dependent Warfarin Pharmacokinetics: Impact of CYP2C9 Genotype on R‐ and S‐Warfarin and Their Oxidative Metabolites,” Journal of Clinical Pharmacology 57, no. 3 (2017): 382–393, 10.1002/jcph.813. 27539372

[24] T. Xun , Z. Lin , X. Wang , et al., “Advanced Oxidation Protein Products Downregulate CYP1A2 and CYP3A4 Expression and Activity via the NF‐κB‐Mediated Signaling Pathway In Vitro and In Vivo,” Laboratory Investigation 101, no. 9 (2021): 1197–1209, 10.1038/s41374-021-00610-9. 34031539 PMC8367815

[25] S. W. Ryter , “Heme Oxygenase‐1: An Anti‐Inflammatory Effector in Cardiovascular, Lung, and Related Metabolic Disorders,” Antioxidants (Basel) 11, no. 3 (2022): 555, 10.3390/antiox11030555. 35326205 PMC8944973

[26] L. Jing , R. Gao , J. Zhang , et al., “Norwogonin Attenuates Hypoxia‐Induced Oxidative Stress and Apoptosis in PC12 Cells,” BMC Complementary Medicine and Therapies 21, no. 1 (2021): 18, 10.1186/s12906-020-03189-8. 33413359 PMC7791982

[27] T. Pecchillo Cimmino , I. Panico , S. Scarano , et al., “Formyl Peptide Receptor 2‐Dependent cPLA2 and 5‐LOX Activation Requires a Functional NADPH Oxidase,” Antioxidants (Basel) 13, no. 2 (2024): 220, 10.3390/antiox13020220. 38397818 PMC10886330

[28] Y. T. Wu , L. Chen , Z. B. Tan , et al., “Luteolin Inhibits Vascular Smooth Muscle Cell Proliferation and Migration by Inhibiting TGFBR1 Signaling,” Frontiers in Pharmacology 9 (2018): 1059, 10.3389/fphar.2018.01059. 30298006 PMC6160560

[29] Y. Luo , P. Shang , and D. Li , “Luteolin: A Flavonoid That Has Multiple Cardio‐Protective Effects and Its Molecular Mechanisms,” Frontiers in Pharmacology 8 (2017): 692, 10.3389/fphar.2017.00692. 29056912 PMC5635727

[30] L. Zhang , Y. Li , W. Yang , et al., “Protocatechuic Aldehyde Increases Pericyte Coverage and Mitigates Pericyte Damage to Enhance the Atherosclerotic Plaque Stability,” Biomedicine & Pharmacotherapy 168 (2023): 115742, 10.1016/j.biopha.2023.115742. 37871558

[31] R. Ji , H. Sun , J. Peng , et al., “Rosmarinic Acid Exerts an Antagonistic Effect on Vascular Calcification by Regulating the Nrf2 Signalling Pathway,” Free Radical Research 53, no. 2 (2019): 187–197, 10.1080/10715762.2018.1558447. 30864863

[32] H. Y. Cui , X. J. Zhang , Y. Yang , et al., “Rosmarinic Acid Elicits Neuroprotection in Ischemic Stroke via Nrf2 and Heme Oxygenase 1 Signaling,” Neural Regeneration Research 13, no. 12 (2018): 2119–2128, 10.4103/1673-5374.241463. 30323140 PMC6199925

