# Supplementary material for: Quality Control of Shenqi Tongmai Oral Liquid Based on Quantitative Analysis of Multicomponents by Single Marker, Molecular Docking, and Multivariate Statistics
Source: Phytochem Anal. 2025 Feb 19;36(5):1450–63. doi: 10.1002/pca.3520 (PMC12212019; doi:10.1002/pca.3520)
Supplement: Supplementary file 1 — Figure S1 HPLC chromatograms of SQTM samples under three different extraction solutions. Figure S2 HPLC chromatograms of SQTM samples in four different mobile phase systems. Figure S3 HPLC chromatograms of SQTM samples at eight different wavelengths. Figure S4 HPLC chromatograms of SQTM samples at three different flow rates. Table S1 Method validation for the simultaneous quantification of the nine constituents in SQTM (n = 6) Figure S5 Influence of different instruments, chromatographic columns, column temperature and flow rates on RCFs. The size of the bubble corresponds to the magnitude of the fi value; a larger bubble indicates a higher fi value. The color of the bubble reflects the RSD value, with a bluer hue signifying a greater RSD. Figure S6 Effect of different instruments, chromatographic columns, column temperatures and flow rates on RRTs. The size of the bubble corresponds to the magnitude of the RRT value; a larger bubble indicates a higher RRT value. The color of the bubble reflects the RSD value, with a bluer hue signifying a greater RSD. Table S2 Contents determination results of nine ingredients by QAMS and ESM (μg/mL) [file PCA-36-1450-s001.docx]

**Quality control of Shenqi Tongmai oral liquid based on** **quantitative analysis of multi-components by single marker, molecular docking, and multivariate statistics**

Qian Li^1,2^ | Li Wang^1,2^ | Liuping Tan^1^ | Xiaojing Tao^1^ | Bei Zhang^1^ | Jingnan Pei^1,2^ |Qiuping Li ^1^

^1^Department of Pharmacy, Liuzhou Hospital of Traditional Chinese Medicine, Liuzhou, China | 2. Graduate School of Guangxi University of Traditional Chinese Medicine, Nanning, China

**Figure S1** | HPLC chromatograms of SQTM samples under three different extraction solutions.

**Figure S2** | HPLC chromatograms of SQTM samples in four different mobile phase systems.

**Figure S3**| HPLC chromatograms of SQTM samples at eight different wavelengths.

**Figure S4** | HPLC chromatograms of SQTM samples at three different flow rates.

**Table S1** | Method validation for the simultaneous quantification of the nine constituents in SQTM (n = 6)

**Figure S5** | Influence of different instruments, chromatographic columns, column temperature and flow rates on RCFs. The size of the bubble corresponds to the magnitude of the fi value; a larger bubble indicates a higher fi value. The color of the bubble reflects the RSD value, with a bluer hue signifying a greater RSD.

**Figure S6** | Effect of different instruments, chromatographic columns, column temperatures and flow rates on RRTs. The size of the bubble corresponds to the magnitude of the RRT value; a larger bubble indicates a higher RRT value. The color of the bubble reflects the RSD value, with a bluer hue signifying a greater RSD.

**Table S2** | Contents determination results of nine ingredients by QAMS and ESM (µg/mL)


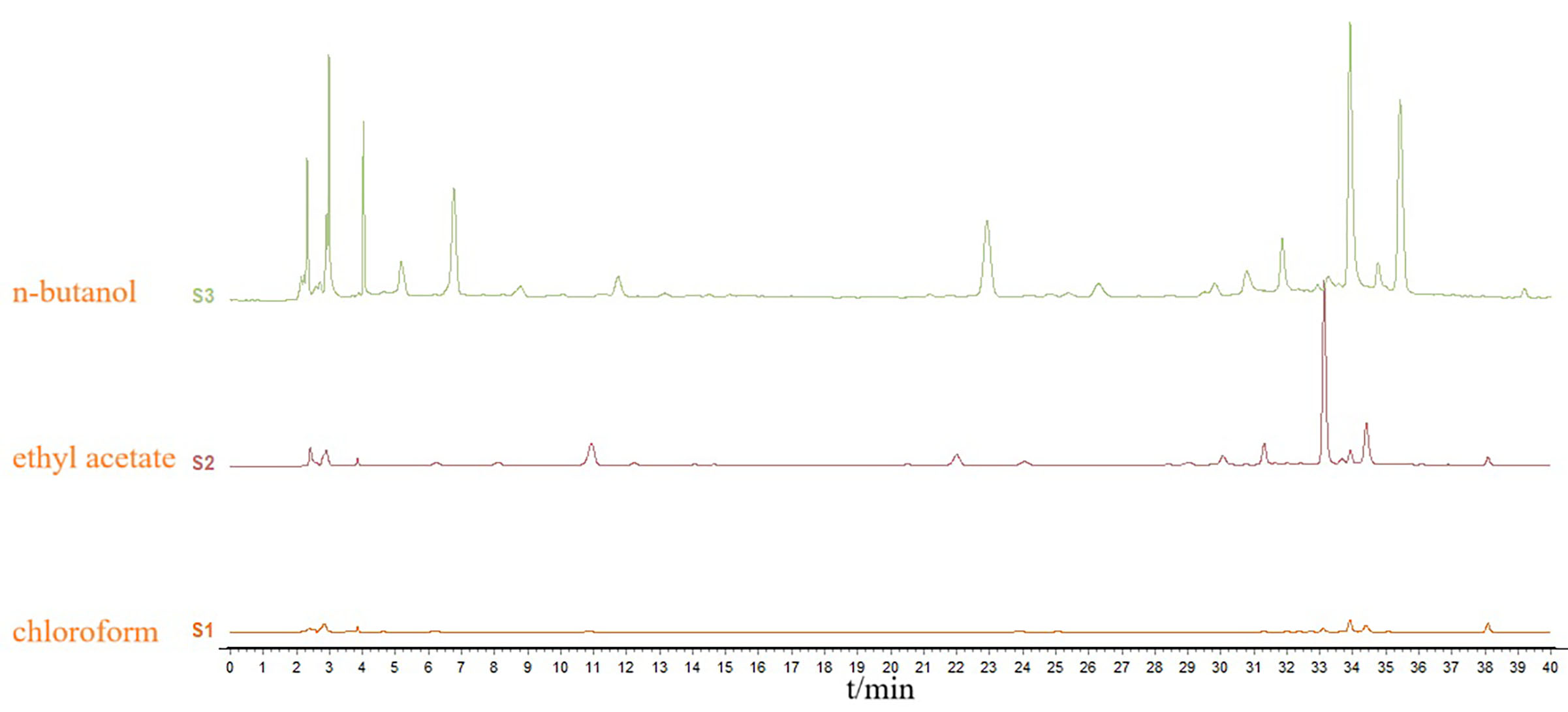


**Figure S1** | HPLC chromatograms of SQTM samples under three different extraction solutions.


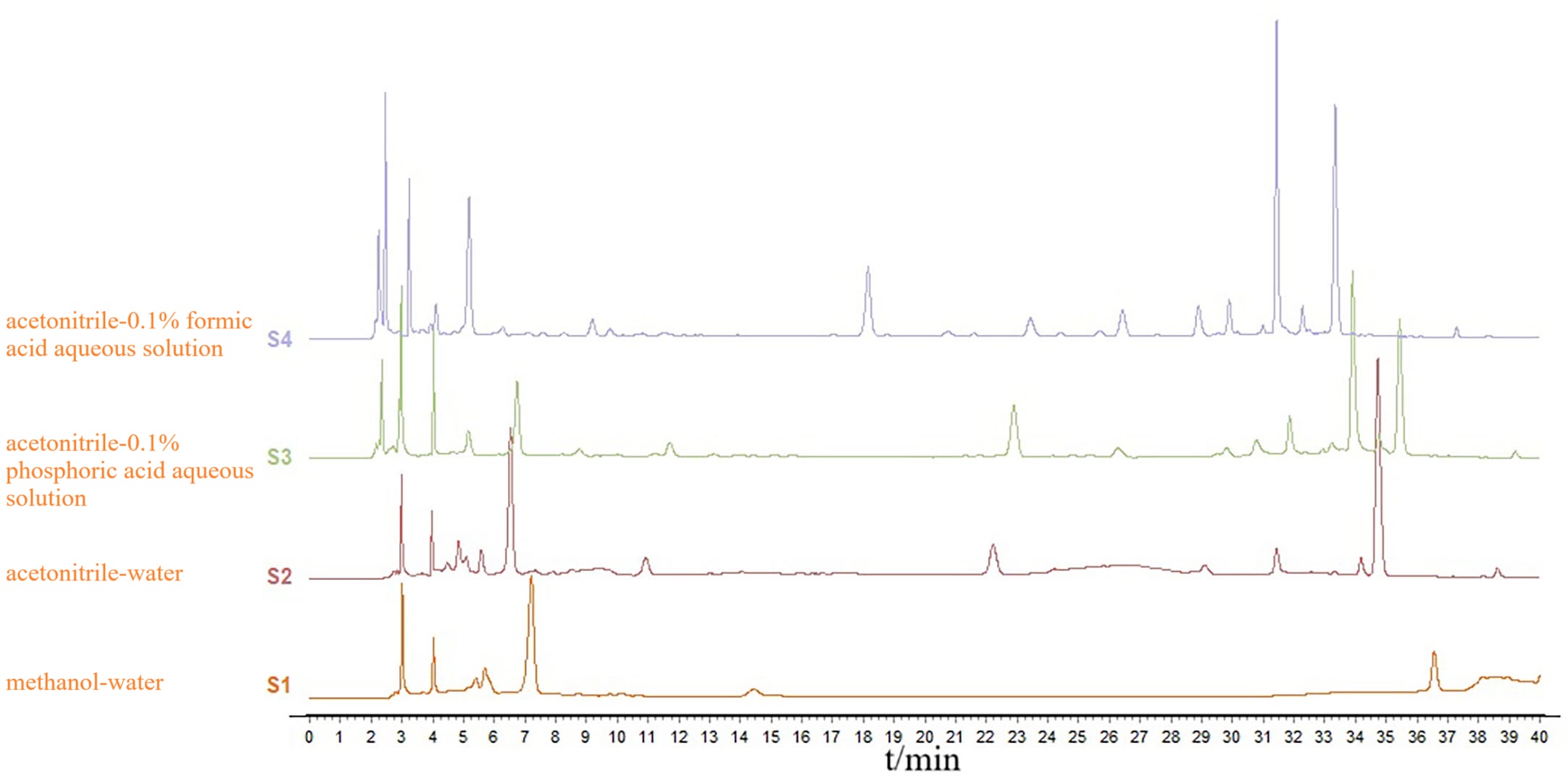


**Figure S2** | HPLC chromatograms of SQTM samples in four different mobile phase systems.


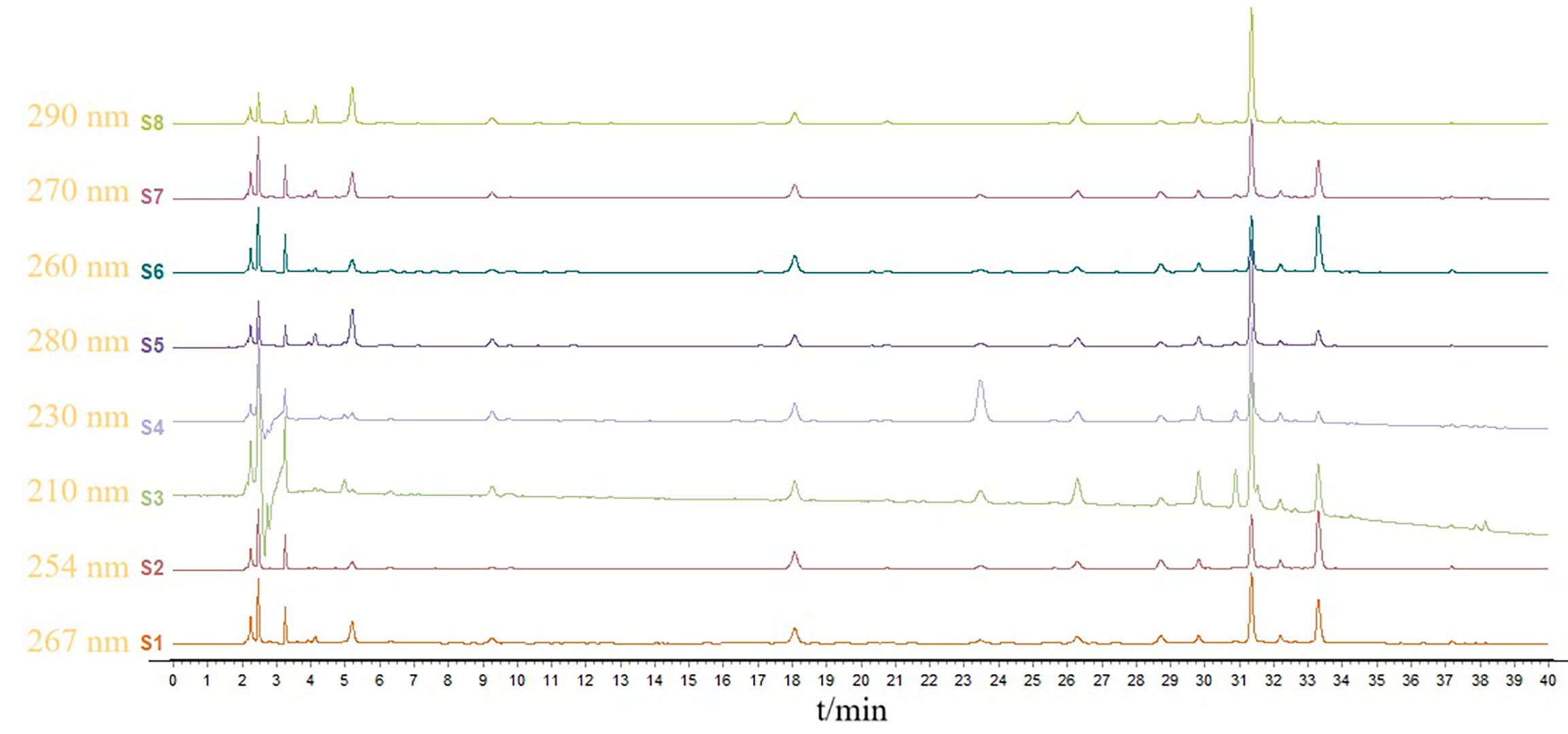


**Figure S3** | HPLC chromatograms of SQTM samples at eight different wavelengths.


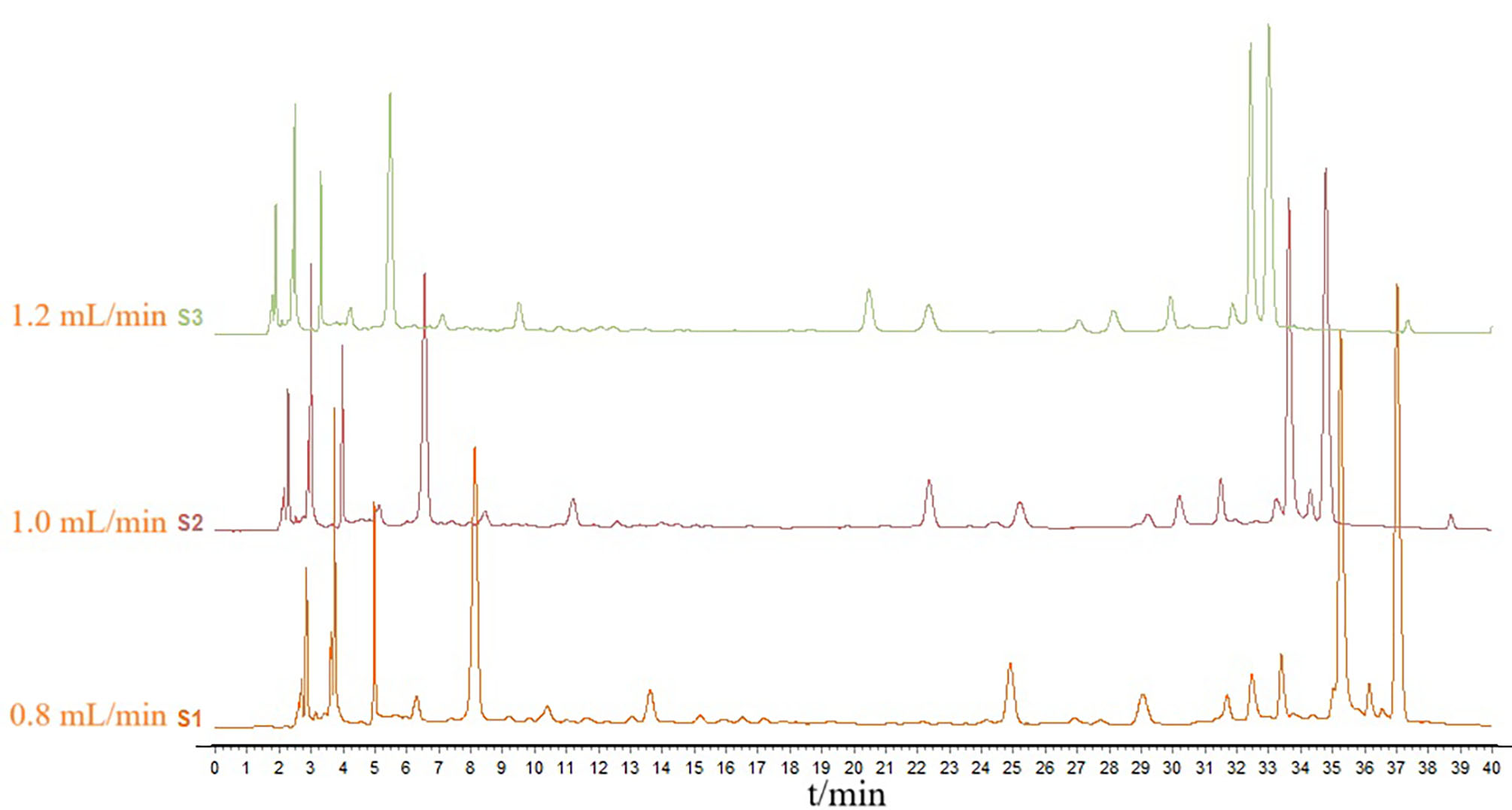


**Figure S4** | HPLC chromatograms of SQTM samples at three different flow rates.

**TABLE S1** | The precision, repeatability, stability, and recovery test of the common peaks in SQTM (n = 6)

| Components | Precision | Repeatability | Stability | Recovery test | |
| --- | --- | --- | --- | --- | --- |
|  |  |  |  | Mean | RSD |
| Protocatechualdehyde | 1.29 | 1.56 | 1.14 | 98.50 | 1.58 |
| Caffeic acid | 2.26 | 1.75 | 2.30 | 100.02 | 2.49 |
| Calycosin-7-O-beta-D-glucoside | 2.35 | 2.31 | 2.42 | 103.08 | 1.37 |
| Naringin | 1.39 | 1.83 | 1.73 | 99.78 | 2.37 |
| Rosmarinic acid | 1.04 | 2.24 | 1.04 | 99.28 | 1.99 |
| Ononin | 1.10 | 1.91 | 1.21 | 97.48 | 0.62 |
| Baicalin | 1.63 | 2.46 | 1.63 | 99.22 | 0.83 |
| Luteolin | 1.01 | 2.20 | 1.10 | 101.31 | 2.04 |
| Formononetin | 2.27 | 2.40 | 2.46 | 97.80 | 1.82 |


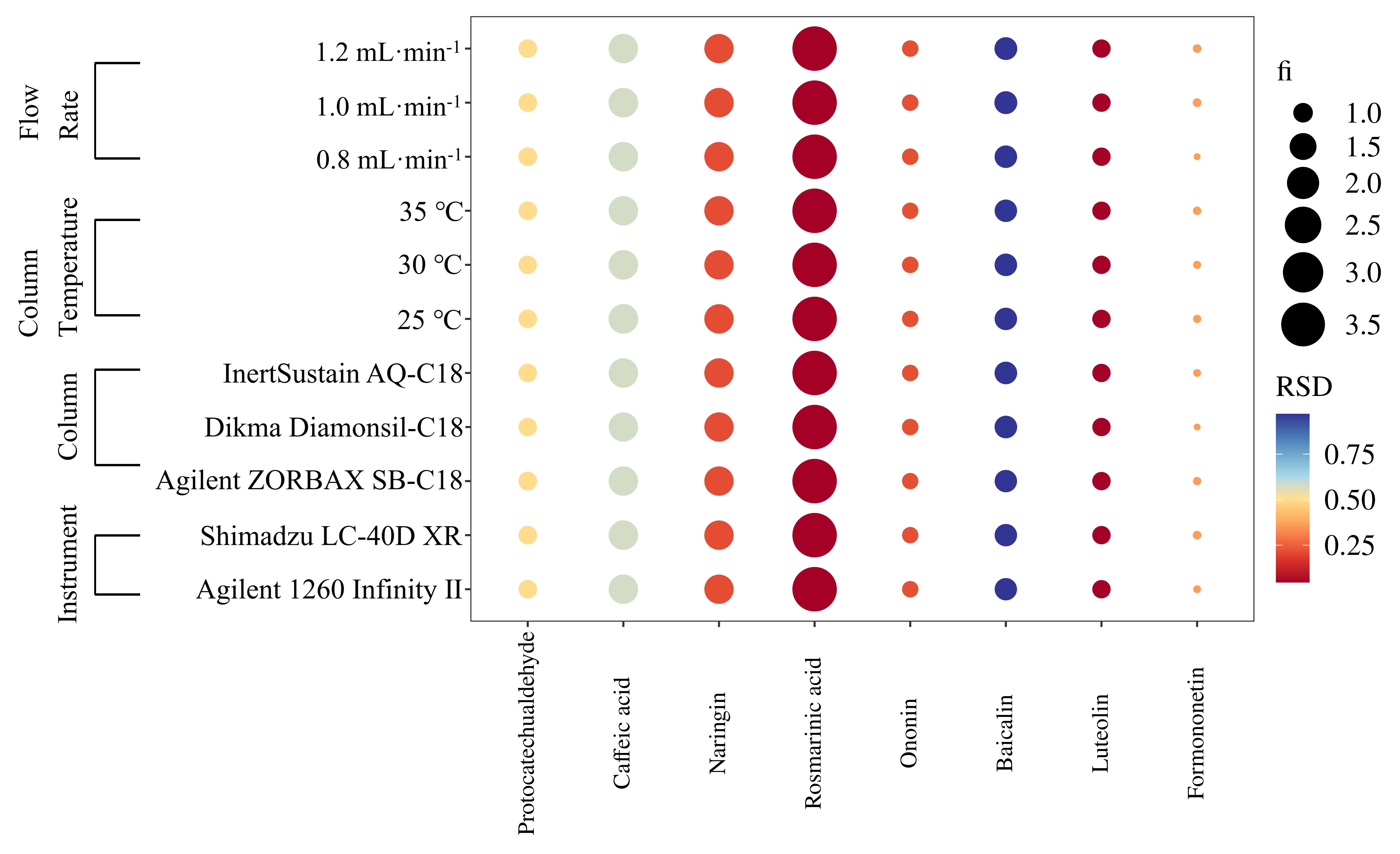


**FIGURE S5** | Influence of different instruments, chromatographic columns, column temperature and flow rates on RCFs. The size of the bubble corresponds to the magnitude of the fi value; a larger bubble indicates a higher fi value. The color of the bubble reflects the RSD value, with a bluer hue signifying a greater RSD.


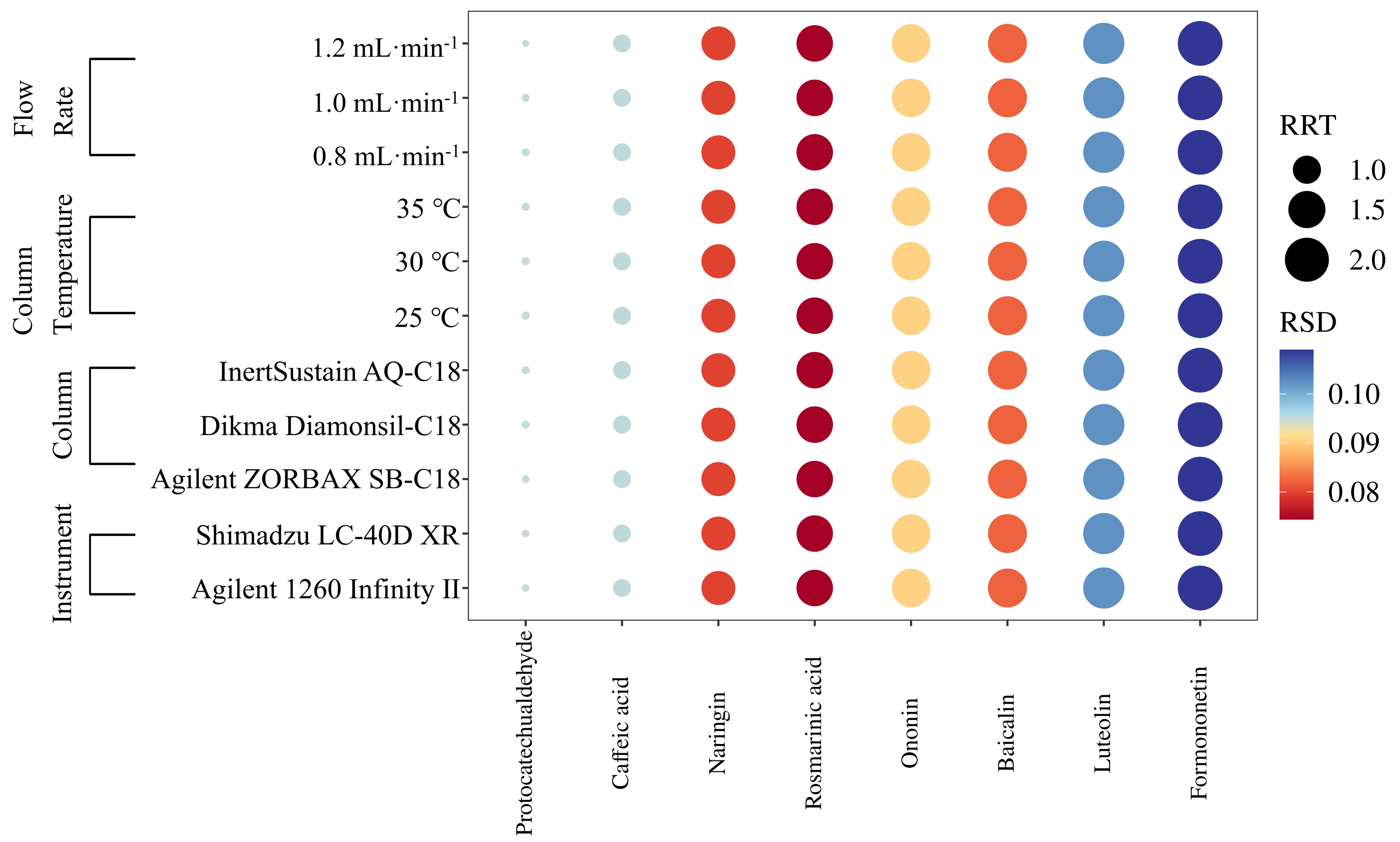


**FIGURE S****6** | Effect of different instruments, chromatographic columns, column temperatures and flow rates on RRTs. The size of the bubble corresponds to the magnitude of the RRT value; a larger bubble indicates a higher RRT value. The color of the bubble reflects the RSD value, with a bluer hue signifying a greater RSD.

**TABLE S2** | Contents determination results of nine ingredients by QAMS and ESM (µg/mL)

| Sample | Calycosin-7-O-beta-D-glucoside | Protocatechualdehyde | | Caffeic acid | | Naringin | |
| --- | --- | --- | --- | --- | --- | --- | --- |
|  | ESM | ESM | QAMS | ESM | QAMS | ESM | QAMS |
| S1 | 31.65 | 12.44 | 12.43 | 4.74 | 4.75 | 12.81 | 12.85 |
| S2 | 36.27 | 19.88 | 19.87 | 5.64 | 5.64 | 25.75 | 25.70 |
| S3 | 31.44 | 19.52 | 19.41 | 4.30 | 4.33 | 16.27 | 16.28 |
| S4 | 34.66 | 14.65 | 14.77 | 4.07 | 4.10 | 15.15 | 15.18 |
| S5 | 34.95 | 18.21 | 18.24 | 4.60 | 4.62 | 20.84 | 20.83 |
| S6 | 35.69 | 19.35 | 19.35 | 10.39 | 10.28 | 24.06 | 24.03 |
| S7 | 42.72 | 15.40 | 15.52 | 6.53 | 6.51 | 20.29 | 20.30 |
| S8 | 33.09 | 19.38 | 19.37 | 4.30 | 4.32 | 18.30 | 18.30 |
| S9 | 33.47 | 24.27 | 24.12 | 5.28 | 5.28 | 19.38 | 19.38 |
| S10 | 50.19 | 26.10 | 25.89 | 9.52 | 9.43 | 50.73 | 50.52 |
| S11 | 40.28 | 18.12 | 18.17 | 6.04 | 6.03 | 16.68 | 16.71 |
| S12 | 75.55 | 28.16 | 27.95 | 7.06 | 7.03 | 26.03 | 26.00 |
| S13 | 40.65 | 19.07 | 19.27 | 6.15 | 6.13 | 13.68 | 13.73 |
| S14 | 76.56 | 18.97 | 19.03 | 6.13 | 6.14 | 21.25 | 21.30 |
| S15 | 83.73 | 16.59 | 16.70 | 4.91 | 4.93 | 26.68 | 26.67 |
| S16 | 72.64 | 20.46 | 20.48 | 14.68 | 14.51 | 49.91 | 49.81 |
| S17 | 52.27 | 22.28 | 22.27 | 13.55 | 13.41 | 35.92 | 35.91 |

**TABLE S2** | (Continued)

| Sample | Rosmarinic acid | | Ononin | | Baicalin | | Luteolin | | Formononetin | |
| --- | --- | --- | --- | --- | --- | --- | --- | --- | --- | --- |
|  | ESM | QAMS | ESM | QAMS | ESM | QAMS | ESM | QAMS | ESM | QAMS |
| S1 | 116.19 | 116.11 | 14.14 | 14.16 | 2.02 | 2.05 | 7.96 | 7.96 | 2.93 | 2.94 |
| S2 | 94.31 | 94.34 | 14.62 | 14.64 | 2.27 | 2.29 | 14.04 | 14.03 | 5.75 | 5.72 |
| S3 | 100.20 | 100.14 | 12.56 | 12.61 | 2.16 | 2.18 | 11.17 | 11.16 | 3.96 | 3.96 |
| S4 | 98.94 | 98.94 | 17.58 | 17.56 | 2.81 | 2.80 | 7.66 | 7.66 | 2.61 | 2.62 |
| S5 | 100.67 | 100.67 | 17.40 | 17.38 | 2.85 | 2.84 | 8.63 | 8.63 | 3.04 | 3.05 |
| S6 | 111.87 | 111.88 | 16.37 | 16.37 | 2.33 | 2.34 | 12.15 | 12.15 | 4.09 | 4.09 |
| S7 | 124.70 | 124.80 | 21.68 | 21.62 | 2.66 | 2.66 | 11.14 | 11.15 | 3.86 | 3.86 |
| S8 | 100.20 | 100.17 | 13.00 | 13.04 | 2.35 | 2.36 | 11.38 | 11.38 | 4.20 | 4.19 |
| S9 | 126.81 | 126.74 | 17.59 | 17.56 | 2.57 | 2.57 | 8.24 | 8.24 | 2.58 | 2.59 |
| S10 | 164.81 | 164.86 | 19.73 | 19.70 | 4.78 | 4.70 | 19.78 | 19.77 | 7.27 | 7.24 |
| S11 | 140.54 | 140.60 | 18.89 | 18.86 | 4.17 | 4.11 | 19.31 | 19.30 | 6.89 | 6.86 |
| S12 | 122.41 | 122.53 | 21.08 | 21.03 | 3.73 | 3.69 | 14.80 | 14.80 | 4.41 | 4.40 |
| S13 | 124.68 | 124.75 | 19.66 | 19.63 | 3.62 | 3.58 | 16.49 | 16.50 | 6.14 | 6.12 |
| S14 | 117.26 | 117.61 | 30.23 | 30.11 | 3.71 | 3.68 | 14.35 | 14.38 | 3.33 | 3.35 |
| S15 | 124.66 | 124.87 | 27.00 | 26.89 | 3.60 | 3.56 | 11.36 | 11.38 | 3.56 | 3.57 |
| S16 | 115.87 | 116.20 | 27.81 | 27.71 | 2.90 | 2.90 | 15.76 | 15.80 | 3.52 | 3.53 |
| S17 | 122.12 | 122.51 | 30.74 | 30.62 | 3.01 | 3.00 | 16.30 | 16.35 | 4.75 | 4.75 |
